# Designing a polyvalent vaccine targeting multiple strains of varicella zoster virus using integrated bioinformatics approaches

**DOI:** 10.3389/fmicb.2023.1291868

**Published:** 2023-11-17

**Authors:** Nurul Amin Rani, Abu Tayab Moin, Rajesh Patil, Tanjin Barketullah Robin, Talha Zubair, Nafisa Nawal, Md. Razwan Sardar Sami, Md Masud Morshed, Jingbo Zhai, Mengzhou Xue, Mohabbat Hossain, Chunfu Zheng, Mohammed Abul Manchur, Nazneen Naher Islam

**Affiliations:** ^1^Faculty of Biotechnology and Genetic Engineering, Sylhet Agricultural University, Sylhet, Bangladesh; ^2^Department of Genetic Engineering and Biotechnology, Faculty of Biological Sciences, University of Chittagong, Chattogram, Bangladesh; ^3^Sinhgad Technical Education Society’s, Department of Pharmaceutical Chemistry, Sinhgad College of Pharmacy, Pune, India; ^4^Notre Dame College, Dhaka, Bangladesh; ^5^Department of Pharmacy, International Islamic University Chittagong, Chattogram, Bangladesh; ^6^Key Laboratory of Zoonose Prevention and Control at Universities of Inner Mongolia Autonomous Region, Medical College, Inner Mongolia Minzu University, Tongliao, China; ^7^Department of Cerebrovascular Diseases, The Second Affiliated Hospital of Zhengzhou University, Zhengzhou, China; ^8^Department of Microbiology, Immunology and Infectious Diseases, University of Calgary, Calgary, AB, Canada; ^9^Department of Microbiology, Faculty of Biological Sciences, University of Chittagong, Chattogram, Bangladesh

**Keywords:** varicella zoster virus, polyvalent vaccine, immunoinformatics, molecular dynamics simulations, immune recognition mechanisms

## Abstract

The Varicella Zoster Virus (VZV) presents a global health challenge due to its dual manifestations of chickenpox and shingles. Despite vaccination efforts, incomplete coverage, and waning immunity lead to recurrent infections, especially in aging and immunocompromised individuals. Existing vaccines prevent chickenpox but can trigger the reactivation of shingles. To address these limitations, we propose a polyvalent multiepitope subunit vaccine targeting key envelope glycoproteins of VZV. Through bioinformatics approaches, we selected six glycoproteins that are crucial for viral infection. Epitope mapping led to the identification of cytotoxic T lymphocyte (CTL), helper T lymphocyte (HTL), and B cell linear (LBL) epitopes. Incorporating strong immunostimulants, we designed two vaccine constructs, demonstrating high antigenicity, solubility, stability, and compatibility with Toll-like receptors (TLRs). Molecular docking and dynamics simulations underscored the stability and affinity of the vaccine constructs with TLRs. These findings lay the foundation for a comprehensive solution to VZV infections, addressing the challenges of incomplete immunity and shingles reactivation. By employing advanced immunoinformatics and dynamics strategies, we have developed a promising polyvalent multiepitope subunit vaccine candidate, poised to enhance protection against VZV and its associated diseases. Further validation through *in vivo* studies is crucial to confirm the effectiveness and potential of the vaccine to curb the spread of VZV. This innovative approach not only contributes to VZV control but also offers insights into tailored vaccine design strategies against complex viral pathogens.

## 1 Introduction

Varicella Zoster Virus (VZV), a human herpes virus, poses a significant global health concern due to its dual clinical manifestations: chickenpox (varicella) and shingles (herpes zoster). This highly contagious virus primarily spreads through respiratory droplets and direct contact with vesicular fluid from active lesions, affecting approximately a million individuals in the United States annually (Daulagala and Noordeen, 2018; Lee et al., 2020). VZV is a DNA virus, and several metabolic pathways including nucleotide, lipid, and amino acid biosynthesis are altered due to their effect (Zandi et al., 2022). Prominent VZV variants include the Oka and Dumas strains. The genome of the Dumas strain comprises linear double-stranded DNA consisting of 125,000 base pairs, and it encompasses 70 open reading frames (ORFs). The primary distinction between the Dumas and Oka strains is characterized by base substitutions and variations in eight amino acids. The Oka strain, utilized in the Varicella vaccine, has effectively reduced global chickenpox incidence while investigating the potential of the Dumas strain as a vaccine vector for other infections. Despite vaccination efforts, VZV infections challenge public health due to incomplete vaccine coverage, waning immunity, and shingles reactivation, especially among aging populations and immunocompromised individuals (Cohen et al., 1999; Gomi et al., 2002; Gershon et al., 2015; CDC, 2021). Hence, in this study, we have employed immunoinformatics and extended dynamics simulation approaches to create and assess a polyvalent vaccine capable of targeting the prevailing virulent strains of VZV.

Current varicella vaccines, such as the low-dose live-attenuated Oka (vOka) vaccine, are generally regarded as safe, causing fewer adverse effects in healthy individuals and effectively preventing varicella. However, they may be associated with the reactivation of herpes zoster (Zeng et al., 2021). Declining cell-mediated immunity (CMI) in aging or immunocompromised individuals can trigger latent VZV reactivation as herpes zoster (Gabutti et al., 2019). The genetic diversity of VZV strains in varicella vaccines suggests that attenuation may result from diverse genes, but the precise mechanism remains unclear (Kang et al., 2020). Recombinant Zoster Vaccine (RZV) and Zoster Vaccine Live (ZVL) are accessible options for preventing herpes zoster. They have demonstrated promising results in clinical trials, exhibiting a high level of reactogenicity, efficacy, and safety. Nonetheless, these vaccines have certain limitations, including uncertain long-term efficacy, a decrease in cell-mediated immunity over time, and restricted suitability for immunocompromised individuals. Their efficacy and safety across populations are evaluated (Singh et al., 2020; Dean, 2021). Thus, a polyvalent vaccine covering multiple VZV strains, including Oka and Dumas, and preventing varicella and herpes zoster is crucial.

A polyvalent vaccine would confer active and passive immunity against various VZV strains, reducing reactivation risks and subsequent shingles outbreaks. It would address other VZV-related illnesses, such as measles and rubella, beyond varicella and herpes zoster. While vaccines exist, a polyvalent vaccine would offer broader protection against different virus strains, particularly benefiting immunocompromised individuals at higher risk of severe complications. Moreover, it would alleviate the overall burden of VZV-associated diseases and enhance public health outcomes (Somboonthum et al., 2007; Daudin and Brézin, 2013; Schlingmann et al., 2018). Furthermore, using the Oka strain as a live attenuated vaccine raises concerns about its efficacy against diverse VZV strains. Research reveals at least four distinct VZV strains with varying biological behaviors. Understanding the genetic basis of Oka’s attenuation compared with clinical isolates can illuminate strain differences, aiding our grasp of their properties and transmissibility (Somboonthum et al., 2007). An analysis of the genome reveals variances in 36 nucleotides between the two Oka strains, thereby restricting the effectiveness of existing vaccines. Polyvalent vaccine encompassing strains such as Oka and Dumas could extend protection against VZV and potentially curtail its spread (Tillieux et al., 2008; Oliver et al., 2021).

The integration of immunoinformatics and dynamics approaches holds paramount significance in the design of polyvalent vaccines. These strategies combine computational analyses of immune responses, epitope prediction, and molecular dynamics simulations to decipher antigen–antibody interactions, epitope presentation, and immune recognition mechanisms, enabling the identification of optimal vaccine candidates, precise targeting of conserved regions, prediction of immune responses, and ultimately accelerating vaccine development processes (Somboonthum et al., 2007; Tillieux et al., 2008; Schlingmann et al., 2018). A polyvalent vaccine possessing these advantages addresses significant limitations, such as shingles reactivation in the elderly population and incomplete vaccine coverage. This approach expedites the design of effective vaccines and enhances our understanding of the immune system–virus interactions, paving the way for more tailored and robust vaccine strategies.

Our study employs bioinformatics approaches to design a polyvalent multiepitope subunit vaccine targeting key envelope glycoproteins B, E, I, H, K, and M of the virus. These glycoproteins play pivotal roles in infection and pathogenesis, suggesting their potential as vaccine candidates. Glycoprotein B (gB), a conserved fusion protein, facilitates entry and cell-to-cell spread (Oliver et al., 2021). Glycoproteins E (gE) and I (gI) are essential for VZV entry and replication, though their functions still need to be clarified. Glycoproteins H (gH) and L (gL) form a fusion-supporting heterodimer complex with gB. Glycoproteins K (gK) and M (gM) facilitate cell-to-cell spread and viral propagation. This innovative approach holds the potential for targeted immune responses and improved strategies against VZV (Oliver et al., 2016). The bioinformatics approach enables the precise identification of optimal epitopes that are capable of eliciting the desired immune response. Nevertheless, it is crucial to validate these findings through rigorous *in vivo* studies to confirm their effectiveness. *In silico* studies serve as preliminary analyses, necessitating subsequent *in vivo* validation (Kaushik et al., 2022). By developing a polyvalent multiepitope subunit vaccine targeting key VZV glycoproteins, we strive to pave the way for a comprehensive solution to the challenges of varicella and herpes zoster infections.

## 2 Methods

**Figure 1:**
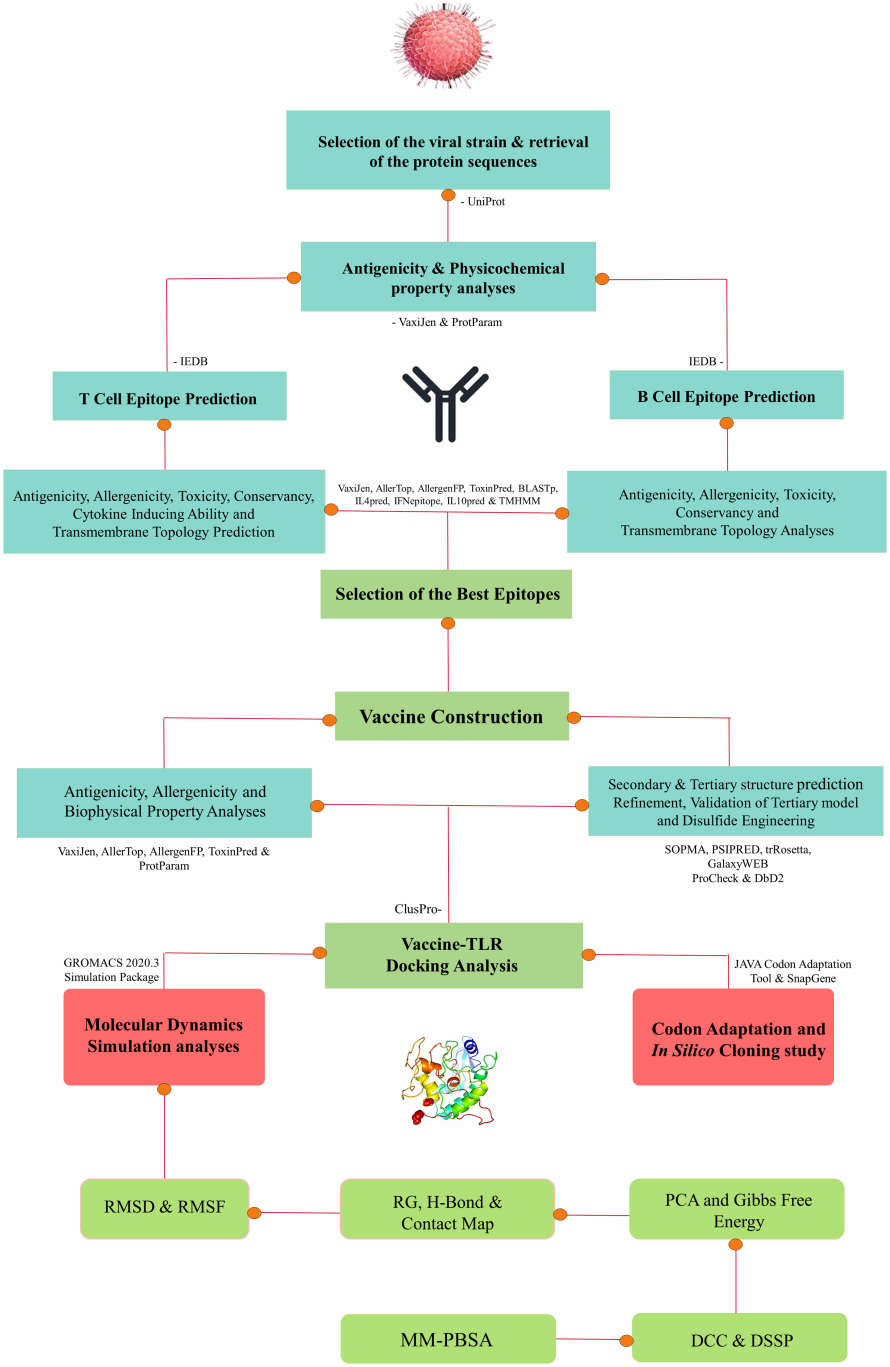
Flowchart presenting the stepwise procedure of the entire study.

The approach employed in this research entails a well-established protocol for conducting immunoinformatics and molecular dynamics analyses, thus enabling a comprehensive exploration of molecular interactions (Sarkar et al., 2021; Araf et al., 2022; Khanam et al., 2022; Moin et al., 2022, 2023a,b; Bappy et al., 2023). The stepwise methods of the entire study are presented as a flowchart in Figure 1.

### 2.1 Selection of strains and proteins with biophysical property analysis

From the *NCBI database*,^1^ two virulent strains of VZV, namely Strain Oka and Strain Dumas, were identified. These strains were chosen for further epitope mapping, focusing on the virulent proteins such as envelope glycoproteins B, E, I, H, K, and M. The target protein sequences of these selected strains were retrieved in FASTA format from the *UniProt* database.^2^ To analyze the conservancy of target proteins between the selected strains, a multiple sequence alignment was generated using *CLC Drug Discovery Workbench 3* software version 3.0 (Biovia et al., 2000). Subsequently, the biophysical properties of the target proteins were analyzed using the Strain Dumas as the model strain. Antigenic properties of the selected protein sequences were assessed through analysis using the *VaxiJen v2.0* server^3^ (Doytchinova and Flower, 2007). The transmembrane topology of the proteins was evaluated using the *TMHMM-2.0* server^4^ (Krogh et al., 2001). Furthermore, other physicochemical characteristics of the proteins were examined using the *ExPASy ProtParam* server^5^ (Gasteiger et al., 2005). Different physicochemical parameters, such as the instability index that determines the stability, the aliphatic index that determines thermostability, and the GRAVY value where a negative score indicates the desired hydrophilicity of the target protein, were evaluated.

### 2.2 Epitope mapping

The prediction of T-cell and B-cell epitopes for the selected protein sequences was carried out using the Immune Epitope Database (IEDB).^6^ Default parameters were maintained during the prediction process. The selection of common HLA alleles for epitope prediction was based on a thorough review of the literature and analyses of previous vaccine studies. For the prediction of MHC class I-restricted CD8+ cytotoxic T-lymphocyte (CTL) epitopes of the selected sequences for some common human leukocyte antigen (HLA) alleles (i.e., HLA A_01:01, HLA A_02:01, HLA A_02:06, HLA A_03:01, HLA A_11–01, and HLA A_29:02), the recommended NetMHCpan EL 4.0 prediction method^7^ was used, with the length of the epitopes set at 9 (9-mer epitopes) for the ease of vaccine development. Similarly, the MHC class II-restricted CD4+ helper T-lymphocyte (HTL) epitopes for some common HLA alleles (i.e., DRB1_03:01, DRB1_04:01, DRB1_15:01, DRB5_01:01, DRB4_01:01, and DRB3_01:01) were determined using the IEDB recommended 2.22 prediction method,^8^ with the length of the epitopes set at 15 (15-mer epitopes). The BepiPred linear epitope prediction method 2.0 was employed to predict the Linear B Lymphocytes (LBL) epitopes of selected proteins, retaining all the default parameters. The top-scored LBL epitopes with suitable attributes were finally selected for further analysis (Kim et al., 2012; Moin et al., 2022; De Groot et al., 2009).

### 2.3 Selection of optimal epitopes for vaccine formulation

After the initial epitope prediction, we evaluated epitope antigenicity using the *VaxiJen v2.0* server. Transmembrane topology was predicted using the *TMHMM 2.0* server. Allergenicity was assessed using the *AllergenFP*,^9^ *AllerTOP*,^10^ and *AlgPred2.0*^11^ servers. Additionally, toxicity was evaluated using the *ToxinPred* server^12^ (Gupta et al., 2013). The capacity of HTL epitopes to induce IFN-γ, IL-4, and IL-10 was predicted using the *IFNepitope*,^13^ *IL4pred*,^14^ and *IL10pred*^15^ servers, respectively (Dhanda et al., 2013a,b; Nagpal et al., 2017). Furthermore, epitope conservancy was evaluated using the *IEDB epitope conservancy* tool.^16^ Epitopes with the highest potential for vaccine construction, based on their high antigenicity, non-toxicity, non-allergenicity, and full conservancy among the targeted strains, were selected. These selected epitopes were integrated into the vaccine design, with adjuvants such as PADRE and human beta-defensin (hBds) and specific linkers, including EAAAK, AAY, GPGPG, and KK.

### 2.4 Analyses of the biophysical and structural properties of the vaccine

Antigenicity and allergenicity analyses were performed to ensure the safety and efficacy of the vaccine. The solubility of the vaccine construct upon expression in Escherichia coli was evaluated using the *Protein-Sol* server^17^ (Hebditch et al., 2017). Additionally, the biophysical characteristics of the vaccine construct, including isoelectric pH, aliphatic and instability indexes, GRAVY values, hydropathicity, anticipated half-life, and other characteristics, were assessed using the *ProtParam* tool of the *ExPASy* server. Preferable isoelectric pH ranges between 4 and 12, an instability index less than 40 is desirable, and a greater aliphatic index indicates a highly thermostable protein (Ikai, 1980; Gamage et al., 2019; Tokmakov et al., 2021). The secondary structure prediction of the multiepitope vaccine construct was performed using two servers, namely, *SOPMA*^18^ and *PSIPRED*^19^ (Geourjon and Deleage, 1995; McGuffin et al., 2000). The tertiary structure of the vaccine constructs was modulated using the *trRosetta* server^20^ and *AlphaFold Protein Structure Database*^21^ followed by refinement using the *GalaxyRefine* module of the *GalaxyWEB* server^22^ (Heo et al., 2013; Du et al., 2021; Jumper et al., 2021). Validation of the refined model was carried out using the *PROCHECK* server^23^ by assessing Ramachandran plots and ERRAT score plots, along with the *ProSA*-web server^24^ for *Z*-score plots (Laskowski et al., 2006; Wiederstein and Sippl, 2007).

### 2.5 Disulfide engineering of the vaccine constructs

Vaccine protein disulfide engineering was performed using the *Disulfide by Design 2* server^25^ to investigate the conformational stability of the folded proteins. Throughout the analysis, the Cα-Cβ-Sγ angle was kept at its default value of 114.6 ± 10, and the *χ*^3^ angle was set at −87° or +97°. Residue pairs with energies lower than 2.5 kcal/mol were chosen and converted to cysteine residues to form disulfide bridges (Craig and Dombkowski, 2013).

### 2.6 Molecular docking analysis

Initially, the binding affinity between the CTL, HTL epitopes, and HLA alleles was investigated using a molecular docking approach. HLA-A*02:01 (PDB ID: 4u6x) for CTL epitopes and HLA-DRB1*15 (PDB ID: 5v4m) for HTL epitopes were chosen for the docking analysis. Both of these HLA alleles are significantly involved with VZV (Pithukpakorn et al., 2015; Tokić et al., 2020). The structure of the HLA alleles was retrieved from the *Protein Database* (PDB) and prepared by *BIOVIA Discovery studio*. The 3D structures of the best CTL and HTL epitopes were generated using the *PEP-FOLD Peptide Structure Prediction* server. The molecular docking analysis was then carried out using the *HDOCK* server^26^ (Yan et al., 2017).

Molecular docking analysis was performed between the vaccine construct and TLR2 (PDB ID: 2z80), TLR3 (PDB ID: 2a0z), and TLR9 (PDB ID: 3wpf) receptors to predict their binding affinities and interaction patterns. The receptor structures of TLR2, TLR3, and TLR9 were obtained from the Protein Database (PDB) and prepared by *BIOVIA Discovery studio*. The ligand was the mutant 3D structure of the vaccine construct obtained from the disulfide engineering analysis. The binding affinity between the vaccine construct and TLRs was calculated using the *ClusPro 2.0* server and the *HDOCK* server (Kozakov et al., 2017). Identifying the best-docked complexes was based on the lowest energy-weighted score and docking efficiency. These complexes were then subjected to extended molecular dynamics simulation studies to further assess the stability of the interactions.

### 2.7 Molecular dynamics simulation studies and MM-PBSA analysis

Molecular dynamics simulations were performed on the docked complexes of TLR2, TLR3, and TLR9 with vaccine construct 1 (V1) and vaccine construct 2 (V2) to assess the stability of corresponding complexes and binding affinity of the vaccine constructs. TLR2 has two chains, chains A and B, while TLR3 and TLR9 have a single chain, chain A. In the docked complexes, the vaccine constructs, V1 and V2, interacted with both the chains of TLR2. We employed the TLR2 complex with both the chains with bound vaccine constructs in MD simulations. MD simulations were performed on six complexes, namely, TLR2-V1, TLR2-V2, TLR3-V1, TLR3-V2, TLR9-V1, and TLR9-V2 using the Groningen Machine for Chemical Simulations (*Gromacs-2020.4*) (Berendsen et al., 1995; Abraham et al., 2015) program on the HPC cluster at Bioinformatics Resources and Applications Facility (BRAF), C-DAC, Pune. The CHARMM-36 force field (Vanommeslaeghe et al., 2010; Best et al., 2012) was used to construct the topologies of the respective protein and vaccine constructs. The respective complexes were solvated with TIP3P water models (Jorgensen and Madura, 1983) in a dodecahedron simulation box with a salt (NaCl) concentration of 0.15 M. The edges of complexes were kept at a distance of 1 nm from the edges of the box. The neutralized solvated systems of each complex were subjected to energy minimization to remove the bad contacts and steric clashes with the steepest descent energy minimization until the force-constant reached the threshold of 100 kJ mol^−1^ nm^−1^. Following the energy minimization, the systems were equilibrated at a constant volume and temperature condition (NVT) where a temperature of 300 K was achieved through a modified Berendsen thermostat (Bussi et al., 2007) for 1 ns and later at a constant volume and pressure condition (NPT) where a pressure of 1 atm was achieved through a Berendsen barostat (Berendsen et al., 1984) for 1 ns. Finally, the unrestrained production phase MD simulations of 100 ns were performed while maintaining the temperature conditions of 300 K with a modified Berendsen thermostat and pressure conditions of 1 atm with the Parrinello–Rahman barostat (Parrinello and Rahman, 1981), with the time step of 2 fs and trajectories stored at each 10 ps. All the covalent bonds were restrained with the Linear Constraint Solve (LINCS) algorithm (Hess et al., 1997), and the long-range electrostatic energies were computed with the Particle Mesh Ewald (PME) method (Petersen, 1995) with a cutoff value of 1.2 nm. The resulting trajectories of MD simulations were first treated for the periodic boundary conditions. The root mean square deviation (RMSD) in the C-α atoms of each TLR chain and chains of vaccine construct in the respective complexes was separately analyzed. Similarly, the root mean square fluctuation (RMSF) in the side chain atoms and the radius of gyrations (Rg) was analyzed for TLR chains and vaccine constructs. The hydrogen bonds formed between the TLR chains and vaccine constructs were analyzed and visually inspected in the intermittent trajectories. The binding free energy between the TLRs and vaccine constructs was approximated through the Poisson–Boltzmann and surface area continuum solvation (MMPBSA) method using the gmx_MMPBSA version 1.52 tool (Valdés-Tresanco et al., 2021).

### 2.8 *In silico* cloning and codon adaptation studies

The *E. coli* strain K12 was chosen as the host for the *in silico* cloning analysis of the vaccine design. *E. coli* is the preferred choice because of its simplicity, rapid and cost-effective high-density production, well-established genetics, and the availability of numerous suitable molecular tools (Fakruddin et al., 2013). However, because the codon usage in humans and *E. coli* differ, the codon adaptation tool *JCAT*^27^ was used to adapt the codon usage of the vaccine construct to well-characterized prokaryotic species to increase the expression rate (Grote et al., 2005). It is critical to avoid bacterial ribosome binding sites, BglII and Apa1 cleavage sites, and Rho-independent transcription termination sites while using the *JCAT* service. The vaccine construct’s optimal sequence was then inverted, followed by conjugation of the N- and C-terminal BglII and Apa1 restriction sites with SnapGene Software.

## 3 Results

### 3.1 Selection of strains and proteins with biophysical property analysis

The target protein sequences in FASTA format were retrieved from the *UniProt* database. Table 1 shows the *UniProt* accession numbers of the selected protein sequences. All the selected proteins were highly antigenic, stable, and have desirable physicochemical properties, as shown in Supplementary Table S1.

**Table 1:** The protein list used in this study with their UniProt accession numbers.

Strain	Isolate No.	Name of the protein	*UniProt* accession No.
Dumas	01	Envelope glycoprotein B	P09257
	02	Envelope glycoprotein E	P09259
	03	Envelope glycoprotein I	P09258
	04	Envelope glycoprotein H	P09260
	05	Envelope glycoprotein K	P09261
	06	Envelope glycoprotein M	P09298
Oka	01	Envelope glycoprotein B	Q4JR05
	02	Envelope glycoprotein E	Q9J3M8
	03	Envelope glycoprotein I	Q77NN4
	04	Envelope glycoprotein H	Q775J3
	05	Envelope glycoprotein K	Q4JQX0
	06	Envelope glycoprotein M	Q77NP2

### 3.2 Epitope mapping and vaccine formulation

**Figure 2:**
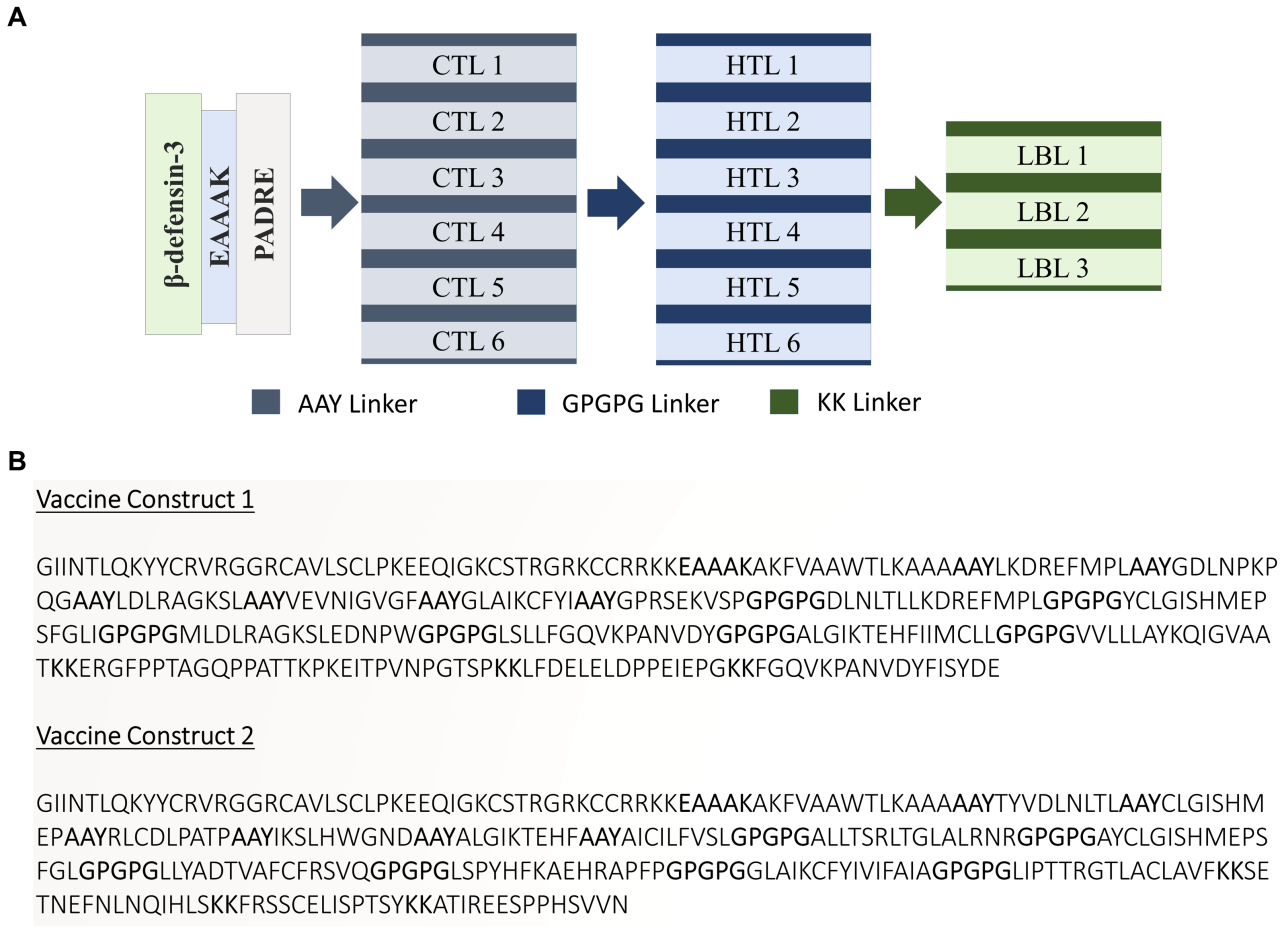
**(A)** Schematic and **(B)** constructive representation of Vaccine construct-V1 and Vaccine construct-V2.

A list of potential CTL, HTL, and LBL epitopes is presented in Supplementary Tables S2–S4. Eventually, 12 CTL, 12 HTL, and 6 LBL epitopes were chosen based on the stringent criteria presented in Supplementary Table S5 for vaccine design. The tools and servers employed to screen the best potential epitopes with their application, input, and output are presented in Supplementary Table S6. Two vaccine constructs were formulated using specific linkers and adjuvants to conjugate the epitopes. Subsequently, these constructs underwent rigorous testing to verify their high antigenicity, non-allergenicity, and non-toxicity. Figures 2A,B present the schematic and constructive representations of vaccine construct-V1 and vaccine construct-V2, respectively.

### 3.3 Analyses of the biophysical and structural properties of the vaccine

Vaccine constructs V1 and V2 exhibited favorable attributes such as solubility, stability, and suitability, which were prerequisites for subsequent experiments (as shown in Supplementary Table S7). Analysis of the secondary structure of the vaccine designs revealed a predominance of the random coil structure. The average Q3 score is predicted to be 76.5% for the secondary structure prediction of PSIPRED server, which is very good (McGuffin et al., 2000). After this, 3D structures were generated for the vaccine constructs, which underwent subsequent refinement and validation. For the best model of trRosetta server, the ERRAT values for V1 and V2 were 98.0392 and 99.6094, respectively, with corresponding *Z*-scores of −1.89 and −2.59. For the best model of AlphaFold, the ERRAT values were 100 and 96.4912, respectively, for V1 and V2 with *Z*-scores of −4.57 and −2.65. Analysis of the Ramachandran plot indicated that most residues in both vaccine designs (for V1 and V2, 98.8 and 96.3% for the trRosetta model and 94.7 and 94.5% for the AlphaFold model, respectively) resided within the favored region.

**Figure 3:**
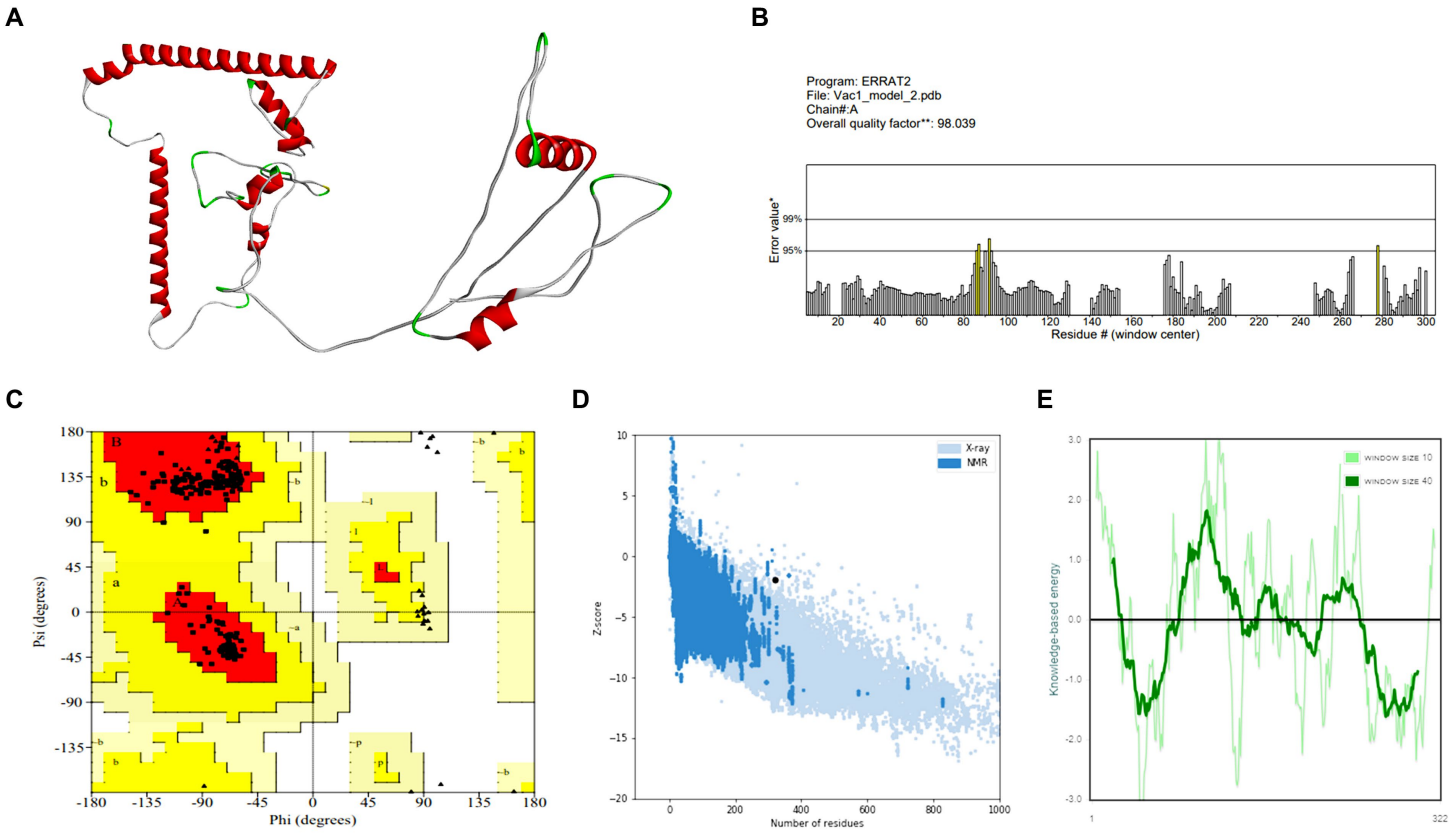
Structure prediction and validation of vaccine construct-V1 **(A)** 3D model, **(B)** The ERRAT quality value, **(C)** Ramachandran plot, **(D)** *Z*-score graph (overall quality), and **(E)** *Z*-score graph (sequence position).

**Figure 4:**
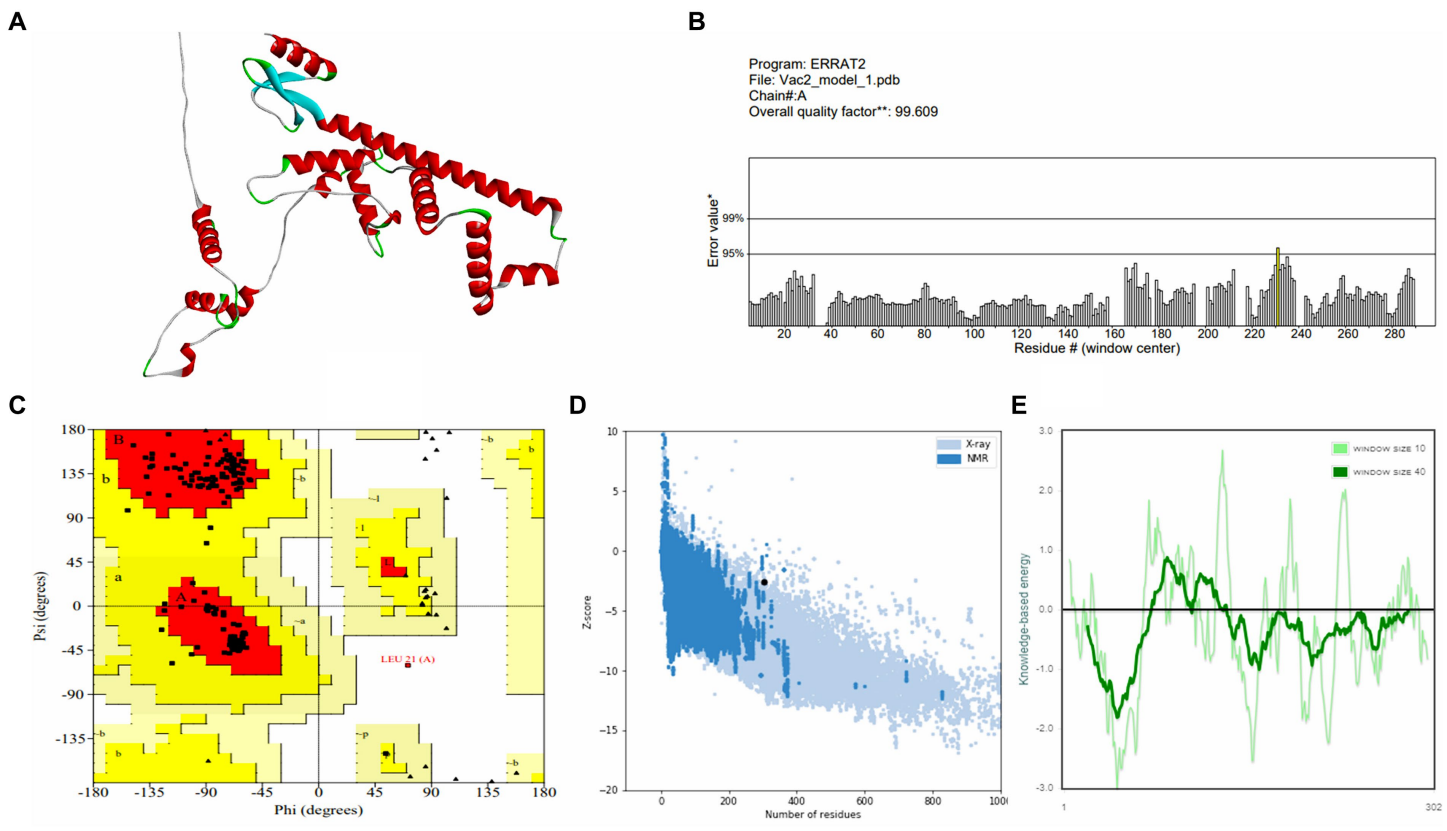
Structure prediction and validation of vaccine construct-V2 **(A)** 3D model, **(B)** The ERRAT quality value, **(C)** Ramachandran plot, **(D)** *Z*-score graph (overall quality), and **(E)** *Z*-score graph (sequence position).

Further analysis of physicochemical properties revealed that vaccine construct-V2 exhibited superior theoretical isoelectric point (9.18 vs. 9.47), Aliphatic index (81.89 vs. 85.13), GRAVY value (−0.169 vs. 0.060), and other characteristics compared with vaccine construct-V1. The collective biophysical and structural features suggested that both vaccine constructs possessed desirable qualities. The 3D models and validation details for both vaccine constructs are presented in Figures 3, 4, respectively. Supplementary information on the secondary structure of the vaccine constructs is presented in Supplementary Table S8 and Figure S1.

### 3.4 Disulfide engineering of vaccine constructs

Using the *DbD2* server, 16 pairs of amino acid residues for vaccine construct-V1 and 13 pairs of amino acid residues for vaccine construct-V2 have been discovered to have the ability to structure disulfide bonds. Three pairs of amino acid residues for vaccine construct-V1 (GLY 15 – ALA 51, SER 34 – GLY37, and ALA 253 – ALA 270) and three pairs of residues for vaccine construct-V2 (CYS 11 – CYS 40, THR 245 – THR 248, and ASN 264 – GLN 267) were carefully selected due to their compatibility with standard disulfide bond formation conditions, with energy levels lower than 2.5 kcal/mol (Supplementary Figures S2, S3).

### 3.5 Molecular docking analysis

The interaction between CTL and HTL epitopes used in vaccine construction with HLA-A*02:01 and HLA-DRB1*15 was assessed through docking analysis, where the epitopes exhibited promising binding affinity (Supplementary Table S9). Subsequently, molecular docking analysis was performed to evaluate the binding affinity of both vaccine constructs with TLR2, TLR3, and TLR9. The results indicated that vaccine construct-V1 exhibited a significantly higher free binding energy and yielded higher docking scores with both TLR2 and TLR9. On the other hand, vaccine construct-V2 exhibited a strong binding affinity toward TLR3. The docking scores obtained from the *ClusPro and HDOCK* servers are presented in Table 2. Based on the assigned docking score, solubility considerations, and other desired criteria, both vaccine constructs were chosen for additional molecular dynamics simulations using the Gromacs 2020.4 package. The simulations aimed to assess further the interaction and binding affinity of the vaccine constructs with TLR2, TLR3, and TLR9.

**Table 2:** Binding affinity between vaccine molecules and TLRs by the ClusPro and HDOCK servers.

TLRs	Vaccine	ClusPro Docking Score (kcal/mol)	HDOCK Docking Score (kcal/mol)
TLR2	V1	−1005.2	−292.86
	V2	−974.1	−288.36
TLR3	V1	−1026.3	−377.57
	V2	−1290.8	−356.74
TLR9	V1	−1337.7	−358.13
	V2	−1161.7	−430.55

A greater negative score indicates high binding affinity in this docking study.

### 3.6 Molecular dynamics studies

#### 3.6.1 Root mean square deviation

**Figure 5:**
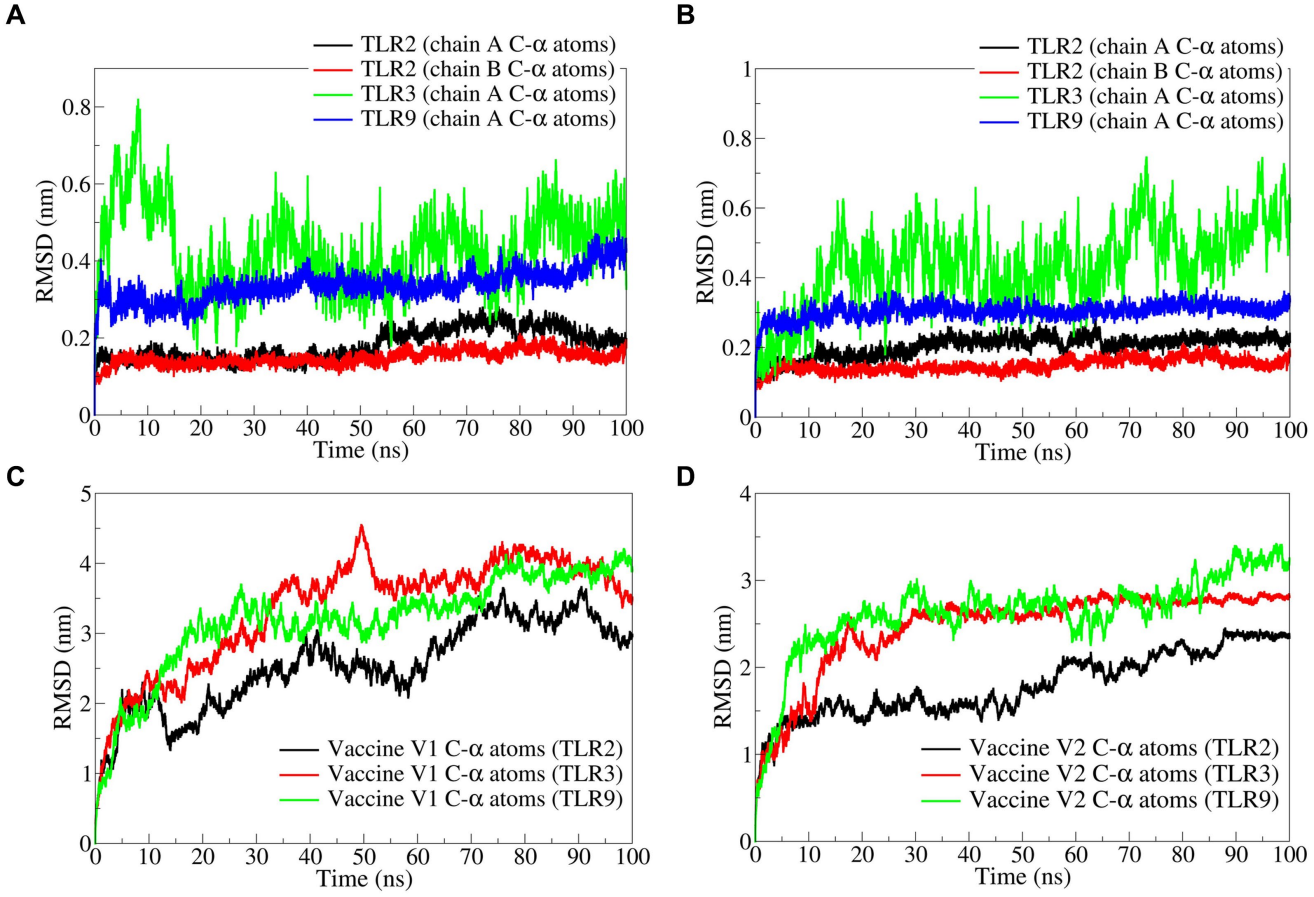
The RMSD analysis. RMSD in C-α atoms of **(A)** TLRs bound to vaccine construct-V1, **(B)** TLRs bound to vaccine construct-V2, **(C)** vaccine construct-V1, and **(D)** vaccine construct-V2.

The RMSD in TLR complexes bound to vaccine construct-V1 showed significant deviations in the TLR3 C-α atoms reaching a maximum of 0.8 nm at approximately 10 ns and an average of 0.4309 nm (Figure 5A and Table 3). Comparably, the RMSD in the TLR9 C-α atoms is reasonably stable with an average of 0.3360 nm. The RMSD in both the chains of TLR2 was considerably lower compared with TLR2 and TLR9, with averages of 0.1815 and 0.2037 nm for chain A and chain B, respectively.

**Table 3:** Estimates of averages for different MDS analysis parameters.

Details of the complexes	Average (nm)						
	RMSD in C-α atoms (TLR chains)	RMSD in C-α atoms (Vaccine chain)	RMSF (TLR chains)	RMSF (Vaccine chains)	Gyrate (TLR chains)	Gyrate (Vaccine chains)	Number of hydrogen bonds
Vaccine construct-V1							
TLR2 (Chain A)	0.1815 (0.0403)	2.5483 (0.6206)	0.0946 (0.0381)	1.5666 (0.4593)	2.3335 (0.0172)	4.2606 (0.6157)	6.50 (2.55)
TLR2 (Chain B)	0.2037 (0.0294)		0.0928 (0.0350)		2.3471 (0.0135)		8.58 (2.02)
TLR3 (Chain A)	0.4309 (0.1025)	3.3534 (0.7955)	0.1524 (0.0748)	1.2488 (0.4465)	3.2935 (0.0496)	3.6631 (0.5825)	14.56 (3.19)
TLR9 (Chain A)	0.3360 (0.0403)	3.1845 (0.7132)	0.1609 (0.0515)	1.2963 (0.4194)	3.2976 (0.0163)	4.1286 (0.5572)	13.46 (3.33)
Vaccine construct-V2							
TLR2 (Chain A)	0.1646 (0.0170)	1.7929 (0.3771)	0.0965 (0.0305)	0.8900 (0.3257)	2.3400 (0.01183)	2.8537 (0.2904)	1.21 (0.99)
TLR2 (Chain B)	0.1483 (0.01848)		0.0838 (0.0304)		2.3508 (0.0060)		6.23 (2.33)
TLR3 (Chain A)	0.4343 (0.1145)	2.4636 (0.4938)	0.1591 (0.0771)	0.7932 (0.4530)	3.2923 (0.0601)	2.8935 (0.1860)	6.49 (2.81)
TLR9 (Chain A)	0.3036 (0.0221)	2.6266 (0.4814)	0.1143 (0.0441)	1.2275 (0.6199)	3.3150 (0.0114)	4.4894 (0.2484)	9.07 (3.28)

Standard deviations in average values are given in parentheses.

Similarly, the RMSD in TLR complexes bound to vaccine construct-V2 showed significant deviations in TLR3 C-α atoms with an average of 0.4343 nm (Figure 5B). The RMSD in TLR9 C-α atoms is stable and lower, with an average of 0.3036 nm. The RMSD in C-α atoms of both the chains of TLR2 is significantly lower, with averages of 0.1646 and 0.1483 nm for chain A and chain B, respectively.

The RMSD in vaccine construct-V1 bound to TLR3 showed significantly higher deviations with an average of 3.3534 nm (Figure 5C). At the same time, the RMSD is slightly lower in complex with TLR9 with an average of 3.1845 nm. The lowest RMSD was observed with the complex of TLR2 with an average of 2.5483 nm.

On the other hand, the RMSD in vaccine construct-V2 showed significantly lower deviations compared with vaccine construct-V1 (Figure 5D). For instance, the RMSD in vaccine construct-V2 C-α atoms was slightly higher in complex with TLR9 and TLR3 with averages of 2.6266 and 2.4636 nm, respectively. The RMSD in C-α atoms of vaccine construct-V2 was lowest in the complex with TLR2 with an average of 1.7929 nm.

**Figure 6:**
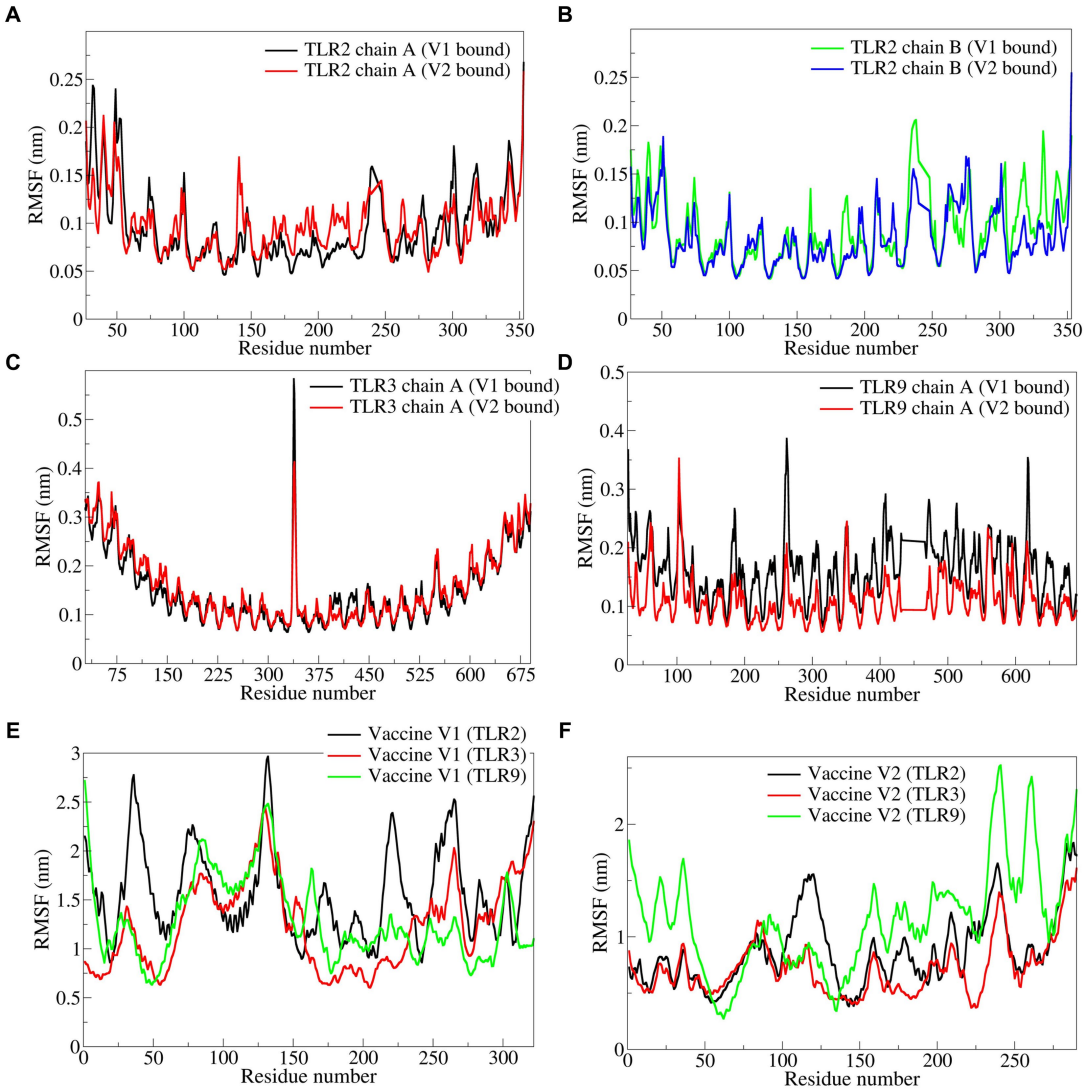
The RMSF in side chain atoms of residues of TLRs and vaccine constructs. **(A)** TLR2 chain A, **(B)** TLR2 chain B, **(C)** TLR3, **(D)** TLR9, **(E)** vaccine construct-V1, and **(F)** vaccine construct-V2.

The RMSF in side chain atoms of TLR chains bound to respective vaccine constructs was analyzed. The RMSF in TLR2 chains A and B was lower in the case of the vaccine construct-V1 bound complex than in the vaccine construct-V2 bound complex. In the case of vaccine construct-V1, the average RMSD values for TLR2 chains A and B were 0.0946 and 0.0928 nm, respectively (Figures 6A,B), while for the vaccine construct-V2, the RMSD values were 0.0965 and 0.0838 nm, respectively. In the case of TLR3 complexes, vaccine construct-V1 had significantly lower RMSF than vaccine construct-V2, where the average for the former was 0.1524 nm and for the latter was 0.1591 nm (Figure 6C). The RMSF in the case of vaccine construct-V2 bound to TLR9 was significantly lower with an average of 0.1143 nm than in the vaccine construct-V2 with an average of 0.1609 nm (Figure 6D). The RMSF in side chain atoms of vaccine constructs bound to TLRs was separately analyzed. The results showed that vaccine construct-V2 had significantly lower RMSF than vaccine construct-V1 in almost all the complexes. Regarding vaccine construct-V1 with TLR2, TLR3, and TLR9, the average RMSF values were 0.0946, 0.1524, and 0.1609, respectively (Figure 6E), while for vaccine construct-V2, the averages for TLR2, TLR3, and TLR9 complexes were 0.0965, 0.0838, and 0.1143 nm, respectively (Figure 6F).

#### 3.6.2 Radius of gyration

**Figure 7:**
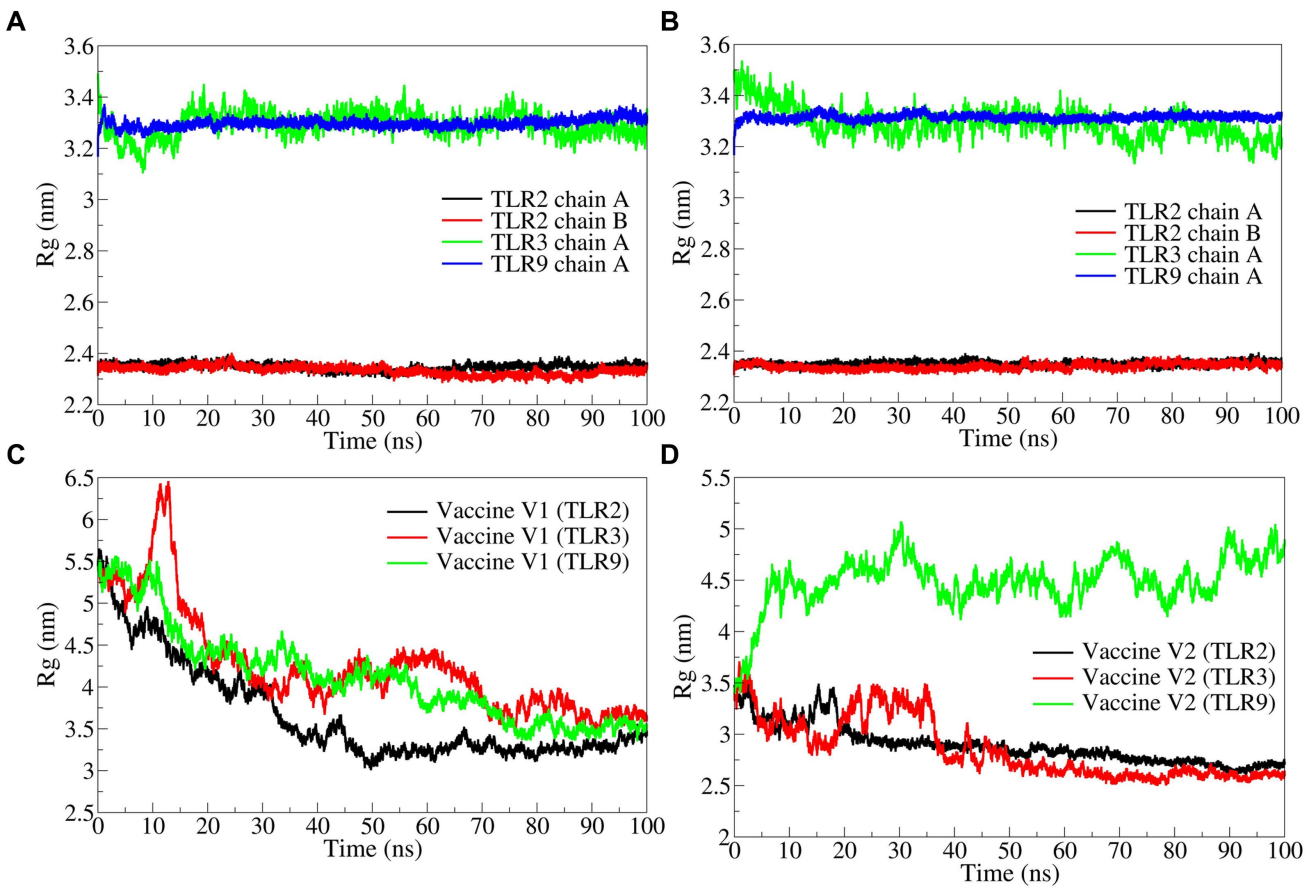
The radius of gyration analysis. **(A)** TLRs in complex with vaccine construct-V1, **(B)** TLRs in complex with vaccine construct-V2, **(C)** Rg in vaccine construct-V1, and **(D)** Rg in vaccine construct-V2.

The total radius of gyration (Rg) for the TLR2 chains was significantly lower in both vaccine construct-V1 and V2 bound complexes with an average Rg range of 2.3400 to 2.3508 nm (Figures 7A,B). At the same time, the deviations were found along with a slightly higher magnitude of Rg in both complexes of TLR3. The Rg in TLR9 complexes was stable but higher compared with TLR2 complexes.

The total Rg for the vaccine constructs was separately analyzed. The results showed that the Rg in vaccine construct-V1 was significantly higher in the TLR2 complex with an average of 4.2606 nm and in the TLR9 complex with an average of 4.1286 nm (Figure 7C). While the vaccine construct-V1 bound to TLR3 had slightly lower Rg with an average of 3.6631 nm. The vaccine construct-V2 bound to TLR2 and TLR3 had significantly lower Rg with averages of 2.8537 and 2.8935 nm, respectively (Figure 7D), whereas, the complex with TLR9 had significantly higher Rg with an average of 4.4894 nm.

#### 3.6.3 Hydrogen bond analysis

**Figure 8:**
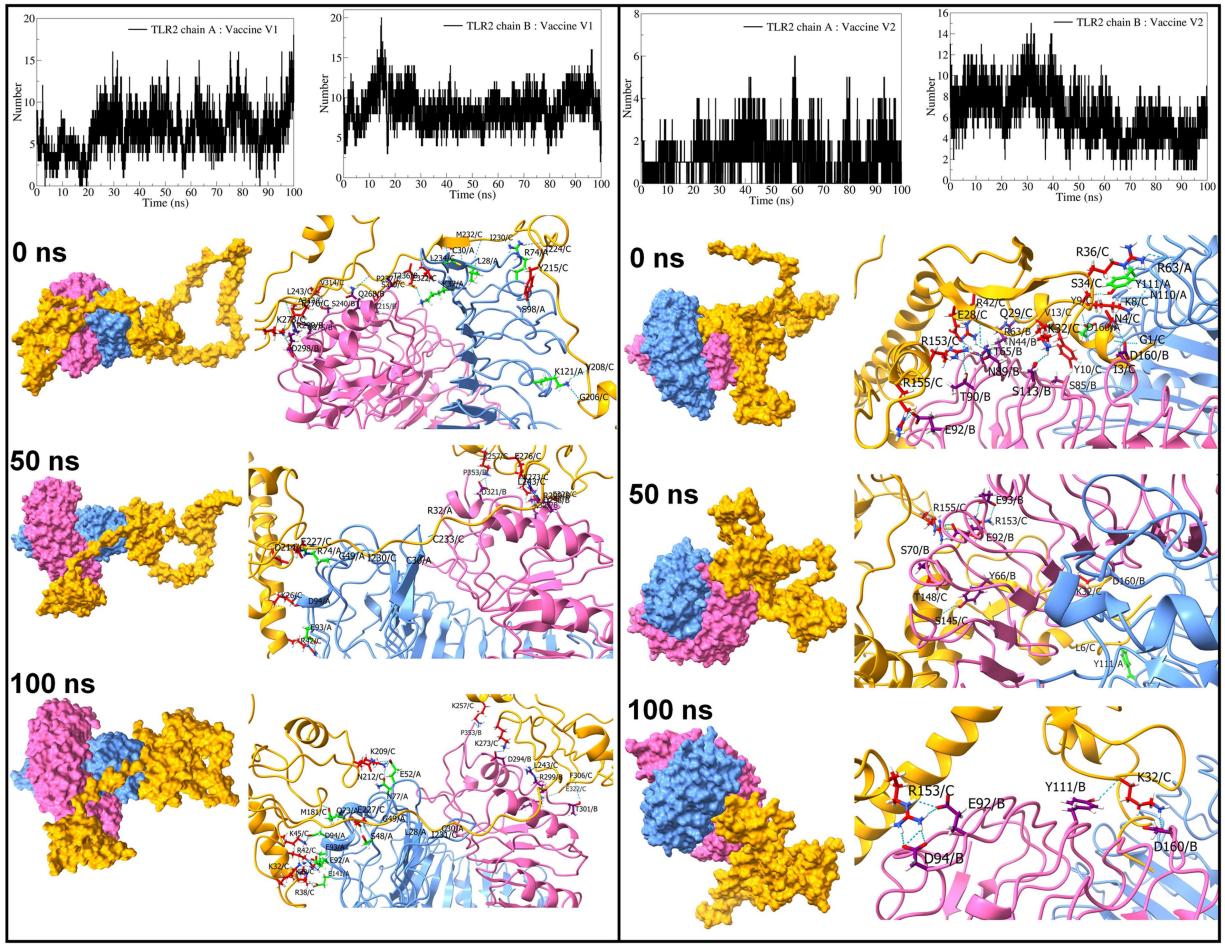
Interchain hydrogen bond analysis for TLR2 and vaccine construct-V1 and vaccine construct-V2 complexes (the surface and cartoon representation of chain A are shown in light blue, chain B in light pink, and vaccine constructs in yellow).

The interchain hydrogen bonds between the TLRs and vaccine constructs were analyzed. The vaccine construct formed approximately 6 hydrogen bonds with TLR2 chain A and approximately 8 hydrogen bonds with TLR2 chain B (Figure 8). In both cases, hydrogen bonds remained stable after approximately 25 ns. The vaccine construct-V2 formed approximately 1 hydrogen bond with TLR2 chain A and approximately 6 hydrogen bonds with chain B. However, after approximately 25 ns, more than 1 hydrogen bond formed between vaccine construct-V2 and TLR2 chain A, and approximately 8 consistent hydrogen bonds were formed until approximately 50 ns, slowly reducing to approximately 6 hydrogen bonds until the end of the simulation. Specifically, the vaccine construct-V1 residues Tyr215, Met232, Leu234, Ala312, Val314, Ser319, Ile230, Glu322, Ile224, Gly206, Val208, Pro237, Leu243, and Glu276 formed hydrogen bonds with TLR2 chain A residues Leu28, Cys30, Lys37, Arg74, Lys121, and Ser98 and chain B residues Thr236, Ser240, Gln268, Arg299, Asp298, Gln275, and Glu215 in the equilibrated trajectory. Most of these hydrogen bonds remained stable throughout the simulation. In the case of vaccine construct-V2, the residues Gly1, Ile3, Asn4, Lys8, Tyr9, Tyr10, Lys32, Arg36, Arg42, Arg153, Arg155, Ser34, Val13, Glu28, and Gln29 formed a hydrogen bond with TLR2 chain A residues Tyr111, Asn110, Asp160, and Arg63 and chain B residues Arg63, Thr65, Asn89, Asp160, Asn89, Ser85, Ser113, Asn44, Thr65, Thr90, and Glu92. Most of these hydrogen bonds remained stable until approximately 50 ns. However, fewer hydrogen bonds were observed after approximately 50 ns.

**Figure 9:**
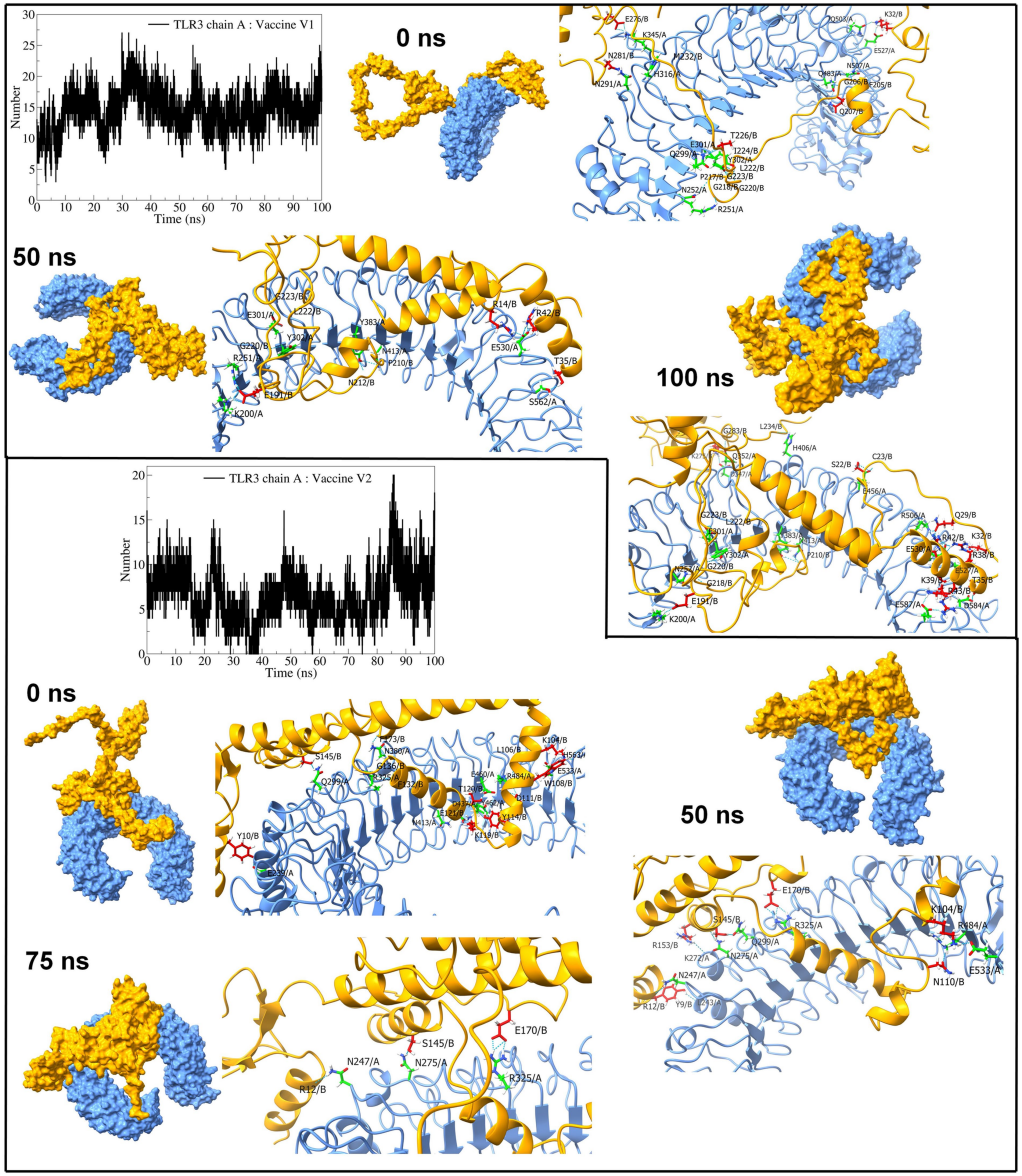
Interchain hydrogen bond analysis for TLR3 and vaccine construct-V1 and vaccine construct-V2 complexes (the surface and carton representation of chain A are shown in light blue and vaccine constructs in yellow).

Regarding TLR3 vaccine construct complexes, vaccine construct-V1 formed an average of 14 hydrogen bonds (Figure 9). However, during the initial 30 ns simulation, fewer hydrogen bonds were formed, which remained consistent after approximately 30 ns. On the other hand, the vaccine construct-V2 formed an average of 6 hydrogen bonds with TLR3. Occasionally, the number of hydrogen bonds was below 5 during the simulation period of 15 ns to 85 ns. Furthermore, the equilibrated trajectory of TLR3 vaccine construct-V1 complex showed hydrogen bonds between the vaccine construct-V1 residues Lys32, Gln207, Ile222, Gly223, Ile224, Asn281, Gln503, Glu527, Gln483, Glu301, and Asn291 and TLR3 residues Arg251, Asn252, Gln299, Tyr302, His316, Lys345, Gln483, and Asn507, while the equilibrated trajectory of TLR3 vaccine construct-V2 complex showed hydrogen bonds between vaccine construct-V2 residues Tyr10, Lys104, Trp108, Tyr114, Lys119, Thr120, Ser145, Phe132, Gly136, Phe173, Glu121, Asp111, and Leu106 and TLR3 residues Gln299, Arg325, Asn380, Asn413, Arg484, Glu239, Glu533, His563, Glu53, Tyr462, Asp437, and Glu460. Most of the hydrogen bonds remained stable until the end of the simulation in both complexes.

**Figure 10:**
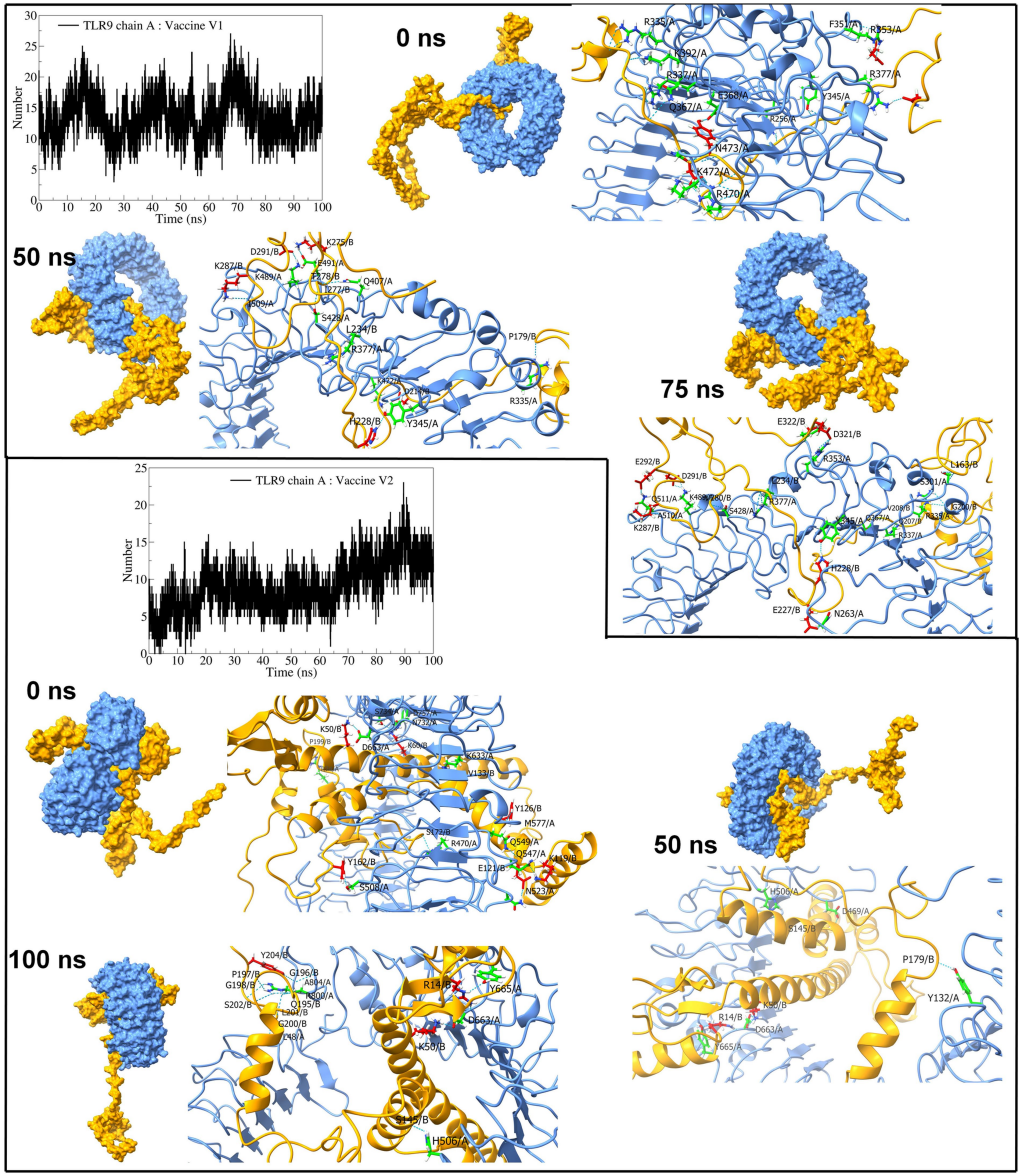
Interchain hydrogen bond analysis for TLR9 and vaccine construct-V1 and vaccine construct-V2 complexes (the surface and carton representation of chain A are shown in light blue and vaccine constructs in yellow).

The interchain hydrogen bonds between the TLR9 and vaccine construct-V1 deviated considerably throughout the simulation, forming an average of 13 hydrogen bonds at 15 ns, 40 ns, and 70 ns, reaching a maximum of 20 hydrogen bonds (Figure 10), while in the TLR9 vaccine construct-V2 complex, an average of 9 hydrogen bonds formed. During the first 10 ns, fewer than 10 hydrogen bonds were formed, steadily rising to approximately 10 hydrogen bonds until 70 ns and rising to approximately 15 hydrogen bonds during the last 30 ns simulation period. The equilibrated trajectory of TLR9 vaccine construct-V1 complex showed intrachain hydrogen bonds between vaccine construct-V1 residues Tyr215, Ile230, Glu227, Ser202, Lys209, Pro210, Ile230, Asp321, Glu322, Ala211, Asn281, Val208, Pro217, and Asp214 and TLR9 residues Arg256, Arg335, Arg337, Tyr345, Phe351, Arg353, Gln367, Arg377, Lys392, Arg470, Lys472, Asn473, Glu368, and Tyr345, while the TLR9 vaccine construct-V2 complex showed interchain hydrogen bonds between vaccine construct-V2 residues Lys50, Lys60, Lys119, Tyr126, Tyr162, Pro199, Ser172, Glu121, and Val133 and TLR9 residues Arg74, Arg470, Asn523, Gln549, Lys633, Asp663, Asn732, Ser734, A2sp757, Gln547, Met577, and Ser508. Most hydrogen bonds remained stable in the TLR9 vaccine construct-V1 complex. However, fewer hydrogen bonds were observed during the 50 ns and 100 ns trajectories of the TLR9 vaccine construct-V2 complex.

#### 3.6.4 MM-PBSA calculation

The trajectories extracted from the last 10 ns simulation period were used in the MM-PBSA calculation. The results of the MM-PBSA calculation are shown in Table 4. The binding free energies (ΔTotal) for vaccine construct-V1 in the complexes with TLR2, TLR3, and TLR9 were −106.02, −141.17, and −114.19 kcal/mol, respectively, while the binding free energies for vaccine construct-V2 in the complexes with TLR2, TLR3, and TLR9 were −65.73, −89.56, and −142.30 kcal/mol, respectively. Vaccine construct-V1 exhibited the lowest electrostatic energies with TLR3, whereas vaccine construct-V2 exhibited the highest electrostatic energies with TLR9. On the contrary, vaccine construct-V1 exhibited the highest polar solvation energies with TLR3, while vaccine construct-V2 exhibited the lowest polar solvation energies.

**Table 4:** MM-PBSA calculations for TLR and vaccine constructs.

Energy component (Kcal/mol)	Vaccine construct-V1			Vaccine construct-V2		
	TLR2	TLR3	TLR9	TLR2	TLR3	TLR9
ΔVDWAALS	−108.06	−182.38	−192.65	−88.35	−137.78	−217.55
ΔEEL	−1546.35	−2207.81	162.60	−1791.22	−1161.31	637.84
ΔEPB	1565.86	2274.98	−58.55	1826.64	1227.71	−535.51
ΔENPOLAR	−17.48	−25.95	−25.59	−12.80	−18.18	−27.08
ΔGGAS	−1654.40	−2390.19	−30.05	−1879.57	−1299.09	420.28
ΔGSOLV	1548.39	2249.02	−84.14	1813.84	1209.53	−562.59
ΔTOTAL	−106.02	−141.17	−114.19	−65.73	−89.56	−142.30

ΔVDWAALS, van der Waals energy; ΔEEL, electrostatic energies; ΔEPB, polar solvation energy; ΔENPOLAR, non-polar solvation energy; ΔGGAS = ΔVDWAALS + ΔEEL; ΔGSOLV = ΔEPB + ΔENPOLAR; ΔTOTAL = ΔGSOLV + ΔGGAS.

### 3.7 *In silico* cloning and codon adaptation studies

The codon adaptation index (CAI) in the vaccine’s codon adaptation study showed that the adapted codons showed a larger percentage of the most prevalent codons. It is noteworthy that the significant GC content of optimized codons is 54.3478 and CAI optimized codons is 1.0. The ideal GC content ranges between 30 and 70% which corresponds to guanine and cytosine content and robustness of the adapted codon (Dana et al., 2020). The CAI value of 1 suggests that the adapted codon is synonymous with the reference set and indicates a good expression level (Puigbo et al., 2009). The absence of restriction sites for SfcI and BanI has been established to ensure the safety of the cloning procedure. The optimized codons were subsequently incorporated into the pET28a (+) vector, along with the SfcI and BanI restriction sites, producing a 2,306-base pair clone containing the desired 827 bp sequence, with the rest belonging to the vector. As shown in Supplementary Figure S4, the desired location between the pET28a (+) vector sequence was highlighted in red.

## 4 Discussion

The human-exclusive neurotropic alphaherpesvirus VZV causes Varicella (chickenpox) and becomes latent in neurons after primary infection. Aging or immunosuppression can lead to reactivation and development of zoster (Brittain et al., 2022; Tayyar and Ho, 2023). VZV is associated with severe complications, such as cancer; effective measures against this virus are urgently needed. Unlike traditional immunization approaches, epitope-based vaccines have advantages, including high specificity, greater safety, ease of manufacture, storage, and long-term efficacy, facilitating the use of epitopes that are capable of conferring protection against numerous strains simultaneously (Banik et al., 2022). Hence, in this study, immunoinformatics and extended dynamics simulations were utilized to design and evaluate a multiepitope polyvalent vaccine against the existing virulent strains of VZV. Six envelope glycoproteins were chosen from two virulent strains due to their role in viral attachment and replication (Oliver et al., 2016). Protein sequences were obtained from UniProt, specifically selecting those with expected antigenic properties and favorable physicochemical characteristics suitable for vaccine formulation.

These proteins were employed for epitope mapping to identify CTL, HTL, and LBL epitopes, which are all important in boosting the immune system of the host in response to viral infection (Shrestha and Diamond, 2004; Chen et al., 2021). IEDB algorithms were used to predict the epitopes, and the epitopes that were ultimately chosen for the vaccine formulation were highly antigenic, non-allergenic, and non-toxic, indicating their ability to elicit a healthy and safe immune response against the viral infection. In total, 12 CTL, 12 HTL, and 6 LBL epitopes were selected based on stringent criteria of antigenicity, non-allergenicity, non-toxicity, and the ability to elicit at least one cytokine response in the case of HTL epitopes. The selected optimal epitopes were finally linked at appropriate positions using EAAAK, AAY, GPGPG, and KK linkers to design two vaccine constructs, namely, V1 and V2. PADRE sequence and hBD adjuvants were integrated to increase their immunogenicity in the human body. The PADRE sequence was utilized as a strong immunostimulant to enhance vaccine potency. The hBD adjuvant promotes innate and adaptive host defense by recruiting immature dendritic cells, naive memory T cells, and monocytes toward the region of infection. EAAAK prevents vaccine degradation, and the introduction of linkers stabilized the vaccine design while increasing antigenicity (Lee and Nguyen, 2015; Moin et al., 2022).

Both vaccine constructs exhibited high antigenicity, non-allergenicity, and non-toxicity, signifying their safety and ability to trigger a robust immune response (Zhang and Tao, 2015). Additionally, both constructs displayed solubility, with a score of 0.507 for V1 and 0.484 for V2. A solubility index exceeding 0.45 is critical, as it indicates higher solubility and reduces the risk of vaccine inefficacy and peptide mass accumulation in the body (Magnan et al., 2009). The physicochemical property analysis demonstrated the proficiency of the vaccine constructs. Both vaccine constructs were stable, with an instability index of 31.42 and 36.12 for vaccine construct-V1 and vaccine construct-V2, respectively. An instability index lower than 40 indicates the stability of the vaccine, whereas a score over 40 describes an unstable vaccine (Gamage et al., 2019). The vaccine construct-V1 appeared to be more stable than vaccine construct-V2 (81.89vs85.13) at normal human body temperature based on the aliphatic index value, which measures thermostability. Such values indicate that thermophilic proteins are substantially more abundant than regular proteins, and the vaccine constructs are thermally more stable (Ikai, 1980). The negative GRAVY value of the vaccine construct-V1 (−0.169) refers to its hydrophilicity, indicating higher water solubility (Kyte and Doolittle, 1982). Additionally, disulfide engineering of the vaccine constructs revealed that the vaccine has multiple pairs of amino acids that can be mutated into cysteine residues, hence improving the stability and efficacy of the vaccine (Gao et al., 2020).

According to secondary structure analysis, a random coil was the most predominant structure in the vaccine designs. After the tertiary structure prediction, the quality of the vaccine models was validated by analyzing the ERRAT and Ramachandran plots. ERRAT refers to the overall quality factor, and a score over 50 suggests the high quality of the model (Messaoudi et al., 2013). The vaccine constructs model showed great ERRAT scores of 98.0392 and 99.6094, indicating high quality. The Ramachandran plot is used to assess the precision of the predicted protein structure. Here, the maximum residue should lie on the favored region, and no residue should reside on the disallowed region in a good plot (Hooft et al., 1997). The Ramachandran plot revealed that the majority of the residues for both vaccine designs (98.8% for vaccine construct-V1 and 96.3% for vaccine construct-V2) were in the favored region and zero to minimum structure in the disallowed region (0% for vaccine construct-V1 and 0.4% for vaccine construct-V2). The overall biophysical and structure prediction analysis indicated that both vaccine designs have adequate properties and structures and should be sufficiently stable.

During the molecular docking study, the binding affinity of the epitopes used in the vaccine with corresponding HLA alleles was assessed. The CTL epitopes showed great binding affinity with MHC1 HLA allele HLA-A*02:01, where the epitope GLAIKCFYI showed the lowest docking score of −184.74 kcal/mol. The HTL epitopes also showed a high binding affinity with MHC2 HLA allele HLA-DRB1*15. The overall analysis revealed the robustness of the epitopes to be used in the vaccine construction. Subsequently, the interaction between both vaccine constructs and TLR2, TLR3, and TLR9 was assessed through molecular docking analysis using the *ClusPro* and *HDOCK* servers. The interaction between vaccines and TLRs is a key indicator for effective protection against infections. TLRs play critical roles in inducing an appropriate immune response (Moin et al., 2022; Salifu et al., 2023). TLR2, TLR3, and TLR9 have been identified as potential targets for the VZV vaccine. TLR2 recognizes viral glycoproteins, including VZV gE, and triggers an immune response (Ciaramella, 2021). TLR3 recognizes viral double-stranded RNA, which is produced during VZV replication, and activates antiviral immune responses (Hanon and Stephenne, 2011). TLR9 recognizes unmethylated CpG motifs in viral DNA, such as VZV DNA, and induces an immune response (Wang et al., 2005; Yu et al., 2011; Kang et al., 2020; Zheng et al., 2020). By targeting these TLRs, VZV vaccines can stimulate both innate and adaptive immune responses, leading to improved protection against VZV infection. Both vaccine constructs exhibited strong affinity with all three TLRs studied. In ClusPro docking analysis, a significantly negative score represents great binding affinity (Kozakov et al., 2017). Vaccine construct-V1 demonstrated the highest binding affinity with TLR9 (−1337.7 kcal/mol), while vaccine construct-V2 showed strong binding with TLR3 (−1290.8 kcal/mol). The docking results of the HDOCK server are more robust than the results of the Cluspro server by showing good binding affinity with all the TLRs, mostly with TLR9 (−358.13 kcal/mol for V1 and −430.55 kcal/mol for V2). The translation efficiency of the vaccine was projected by increasing the mRNA using the Java Codon Adaptation tool and the E. coli strain K-12 as the cell culture system. The final clone had 2,306 base pairs, 827 of which were from the required sequence, while the others were from the vector. This cloned vector has the potential for heterologous cloning and vaccine expression.

Furthermore, extensive high-throughput molecular dynamics simulations were undertaken to delve deeper into the stability of the TLR vaccine construct complexes and the binding affinity of the vaccine constructs. RMSD analysis in protein backbone and ligand atoms gives insights into the stability of the system (Pitera, 2014), while Rg analysis provides insights into the compactness of the systems (Lobanov et al., 2008; Nada et al., 2022). The TLR structure is unique because it contains the structural motifs known as leucine-rich repeat (LRR) of approximately 22–29 residues containing beta-strands and alpha-helices (Botos et al., 2011). Furthermore, being a transmembrane protein, they are several residues long, which confers reasonable stability and a unique horseshoe-shaped structure that can bind to a vaccine or pathogen-associated molecular patterns (Manavalan et al., 2011). The RMSD in TLR2 dimer (chains A and B) and TLR9, bound to vaccine construct-V1 and vaccine construct-V2, is approximately 0.3 nm representing the stability of these complexes. The RMSD in C-α atoms of vaccine constructs is quite high due to flexible, non-compact structure and high content of flexible loop regions. Comparably, the vaccine construct-V1 and vaccine construct-V2 had the lowest RMSD in TLR2 complexes, signifying their potential role in stability.

The RMSF in side chain atoms signifies the fluctuations in its average position and provides valuable information about the conformational changes during the MD simulation (Martínez, 2015; Zhao et al., 2015). Due to the structural features mentioned above, the RMSF in all the studied TLRs showed minor fluctuation below 0.3 nm, signifying their rigid structures. The lowest fluctuations in TLR2 further point out its better stability. The RMSF in vaccine construct-V2 is lower than vaccine construct-V1, which signifies the comparably better stability of vaccine construct-V2. The more significant fluctuations were evident in both vaccine constructs in the loop regions.

The distribution of protein atoms around its axis is measured in terms of the radius of gyration, where the higher the Rg, the more flexibility, representing a less compact and less stable structure (Lobanov et al., 2008). The MD simulations revealed that TLR2 retained its compact structure bound to vaccine constructs, while TLR9 and TLR3 had less compact structures. Both vaccine constructs in complex with TLR2 have significantly lower Rg, implying their compact structure and consequent stability. Comparably, vaccine construct-V2 had lower Rg than vaccine construct-V1 in TLR2 complexes, suggesting better affinity and stability of vaccine construct-V2 over vaccine construct-V1. Similarly, vaccine construct-V2 outperforms TLR3 due to its considerable compact structure after approximately 40 ns. The vaccine construct-V1 remained compact with lower Rg in the TLR9 complex. However, the vaccine construct-V2 having significantly higher Rg suggested its flexible and unstable conformation with TLR9.

The interchain hydrogen bonds confer strong electrostatic interactions between the interacting residues, which is important in holding two proteins together and stability of the resulting protein–protein complex (Yu et al., 2015). Furthermore, the greater the number of interchain hydrogen bonds, the more stable the resulting protein-protein complex is (Pace et al., 2014). The number of interchain hydrogen bonds formed between the TLR2 chains and vaccine construct-V1 is approximately 14, the highest among all the complexes. Furthermore, the vaccine construct-V1 also formed a maximum number of interchain hydrogen bonds with TLR3 and TLR9, signifying better affinity of this vaccine construct. The vaccine construct-V2 also showed reasonable interchain hydrogen bonds, which suggests its potential binding affinity and stability of complexes. In summary, the MD simulations suggested that the vaccine construct-V2 has the strongest propensity of stabilizing the TLR2 and TLR3 complexes. The vaccine construct-V1 has a strong propensity of stabilizing TLR2 and TLR9 complexes.

The binding free energies estimated from the MM-PBSA calculation suggested the strong binding affinity of vaccine construct-V1 with the TLR3 with the ΔTotal of −141.17 kcal/mol. The ClusPro docking score of −1026.3 for vaccine construct-V1 with TLR3 corroborated the MM-PBSA binding free energy. However, for vaccine construct-V1, the binding free energy (ΔTotal = −114.19 kcal/mol) was slightly higher for TLR9, whereas the corresponding docking score was −1337.7. The significant difference between MM-PBSA binding free energy and docking score might have arisen due to significantly higher electrostatic energy contribution and lower polar solvation energy contribution in the TLR-V1 complex. In the case of TLR2-V1 complex, vaccine construct-V1 has comparatively higher binding free energy and docking score compared with the complexes of TLR3 and TLR9 with V1. In the case of vaccine construct-V2, it has the lowest MM-PBSA binding free energy estimates (ΔTotal = −142.30 kcal/mol) and a ClusPro docking score of −1161.7. However, in the case of TLR3, it showed slightly higher binding free energy (ΔTotal = −89.56 kcal/mol) compared with the significantly lower docking score of −1290.8. The lower electrostatic energy and higher polar solvation energy contributions might have resulted in slightly higher binding free energy for the TLR3 complex. Overall, the MM-PBSA binding free energy calculation supports the stability assessment of respective TLR vaccine construct complexes in terms of RMSD, RMSF, Rg, and hydrogen bond analysis.

## 5 Conclusion

VZV presents a global health challenge through chickenpox and shingles. Although current vaccines provide partial protection, the necessity for a polyvalent vaccine spanning multiple VZV strains is evident, particularly for enhanced immunity in vulnerable populations. The integration of immunoinformatics and dynamics in vaccine design holds potential by accelerating candidate identification and predicting immune responses. Our research contributes to more effective strategies against VZV infections, with the goal of bolstering defense against varicella and herpes zoster, thus propelling vaccine development.

## Data availability statement

The original contributions presented in the study are included in the article/Supplementary material, further inquiries can be directed to the corresponding authors.

## Author contributions

NA: Formal analysis, Methodology, Visualization, Writing – original draft, Writing – review & editing. AM: Conceptualization, Data curation, Formal analysis, Funding acquisition, Investigation, Project administration, Visualization, Writing – original draft, Writing – review & editing. RP: Methodology, Software, Visualization, Writing – original draft, Writing – review & editing. TB: Methodology, Visualization, Writing – original draft, Writing – review & editing. TZ: Funding acquisition, Writing – original draft. NN: Funding acquisition, Writing – review & editing. MS: Formal analysis, Visualization, Writing – original draft. MM: Writing – review & editing. JZ: Writing – review & editing. MX: Writing – review & editing. MH: Supervision, Writing – review & editing. CZ: Writing – review & editing. MA: Supervision, Writing – review & editing. NI: Supervision, Writing – review & editing.

## Footnotes

^1^ https://www.ncbi.nlm.nih.gov/

^2^ https://www.uniprot.org

^3^ http://vaxijen/VaxiJen/VaxiJen.html

^4^ https://services.healthtech.dtu.dk/service.php?TMHMM-2.0

^5^ https://web.expasy.org/protparam//

^6^ https://www.iedb.org/

^7^ http://tools.iedb.org/mhci/

^8^ http://tools.iedb.org/mhcii/

^9^ https://ddg-pharmfac.net/AllergenFP/

^10^ https://www.ddg-pharmfac.net/AllerTOP/

^11^ https://webs.iiitd.edu.in/raghava/algpred2/ige.html

^12^ http://crdd.osdd.net/raghava/toxinpred/

^13^ http://crdd.osdd.net/raghava/ifnepitope/

^14^ http://crdd.osdd.net/raghava/il4pred/

^15^ http://crdd.osdd.net/raghava/IL-10pred/

^16^ http://tools.iedb.org/conservancy/

^17^ https://protein-sol.manchester.ac.uk/

^18^ https://npsa-prabi.ibcp.fr/cgi-bin/npsa_automat.pl?page=/NPSA/npsa_sopma.html

^19^ http://bioinf.cs.ucl.ac.uk/psipred/

^20^ https://yanglab.nankai.edu.cn/trRosetta/

^21^ https://alphafold.ebi.ac.uk/

^22^ http://galaxy.seoklab.org/

^23^ https://saves.mbi.ucla.edu/

^24^ https://prosa.services.came.sbg.ac.at/prosa.php

^25^ http://cptweb.cpt.wayne.edu/DbD2/

^26^ http://hdock.phys.hust.edu.cn/

^27^ http://www.jcat.de
