# Corrigendum: Designing a polyvalent vaccine targeting multiple strains of varicella zoster virus using integrated bioinformatics approaches

**DOI:** 10.3389/fmicb.2024.1498557

**Published:** 2024-10-08

**Authors:** Nurul Amin Rani, Abu Tayab Moin, Rajesh Patil, Tanjin Barketullah Robin, Talha Zubair, Nafisa Nawal, Md. Razwan Sardar Sami, Md Masud Morshed, Jingbo Zhai, Mengzhou Xue, Mohabbat Hossain, Chunfu Zheng, Mohammed Abul Manchur, Nazneen Naher Islam

**Affiliations:** ^1^Faculty of Biotechnology and Genetic Engineering, Sylhet Agricultural University, Sylhet, Bangladesh; ^2^Department of Genetic Engineering and Biotechnology, Faculty of Biological Sciences, University of Chittagong, Chattogram, Bangladesh; ^3^Sinhgad Technical Education Society's, Department of Pharmaceutical Chemistry, Sinhgad College of Pharmacy, Pune, India; ^4^Notre Dame College, Dhaka, Bangladesh; ^5^Department of Pharmacy, International Islamic University Chittagong, Chattogram, Bangladesh; ^6^Key Laboratory of Zoonose Prevention and Control at Universities of Inner Mongolia Autonomous Region, Medical College, Inner Mongolia Minzu University, Tongliao, China; ^7^Department of Cerebrovascular Diseases, The Second Affiliated Hospital of Zhengzhou University, Zhengzhou, China; ^8^Department of Microbiology, Immunology and Infectious Diseases, University of Calgary, Calgary, AB, Canada; ^9^Department of Microbiology, Faculty of Biological Sciences, University of Chittagong, Chattogram, Bangladesh

**Keywords:** varicella zoster virus, polyvalent vaccine, immunoinformatics, molecular dynamics simulations, immune recognition mechanisms

In the published article, there was an error in the **Acknowledgments** statement. In the Acknowledgment, we mentioned several individuals and organizations as a token of appreciation. However, this might lead readers to mistakenly believe that these individuals and organizations funded our study. We want to make it clear that our study did not receive any funding from any internal or external sources. The correct Acknowledgment statement appears below.

