# Supplementary material for: Designing a polyvalent vaccine targeting multiple strains of varicella zoster virus using integrated bioinformatics approaches
Source: Front Microbiol. 2023 Nov 17;14:1291868. doi: 10.3389/fmicb.2023.1291868 (PMC10704101; doi:10.3389/fmicb.2023.1291868)
Supplement: Supplementary file 1 [file Data_Sheet_1.PDF]

Supplementary Tables

Supplementary Table S1: The antigenicity and physicochemical property analysis of the selected viral proteins. AN; antigenicity, pI; theoretical pI, II; instability index, AI; aliphatic index, GRAVY; grand average of hydropathicity.

**Virus Strain**	**Protein Name**	**UniProt accession number**	**AN (0.40<)**	**pI (4<)**	**Estimate d half life**	**II (40>)**	**AI (60<)**	**GRAVY**
**Dumas**	Envelope glycoprotein B	P09257	0.5085	8.81	30 hours (mammalian reticulocytes, in vitro).<br><br>>20 hours (yeast, in vivo).<br><br>>10 hours (Escherichia coli, in vivo).	40.73	78.31	-0.351
	Envelope glycoprotein E	P09259	0.5104	5.27	30 hours (mammalian reticulocytes, in vitro).<br><br>>20 hours (yeast, in vivo).<br><br>>10 hours (Escherichia coli, in vivo).	34.90	74.59	-0.392
	Envelope glycoprotein I	P09258	0.4192	8.20	30 hours (mammalian reticulocytes, in vitro).<br><br>>20 hours (yeast, in vivo).<br><br>>10 hours (Escherichia coli, in vivo).	37.26	100.45	-0.022
	Envelope glycoprotein H	P09260	0.5135	6.16	30 hours (mammalian reticulocytes, in vitro).<br><br>>20 hours (yeast, in vivo).	33.93	95.91	0.049

					>10 hours (Escherichia coli, in vivo).			
	Envelope glycoprotein K	P09261	0.5731	9.01	30 hours (mammalian reticulocytes, in vitro).<br><br>>20 hours (yeast, in vivo).<br><br>>10 hours (Escherichia coli, in vivo).	40.35	106.29	0.459
	Envelope glycoprotein M	P09298	0.4934	9.20	30 hours (mammalian reticulocytes, in vitro).<br><br>>20 hours (yeast, in vivo).<br><br>>10 hours (Escherichia coli, in vivo).	36.38	104.44	0.277
**Oka**	Envelope glycoprotein B	Q4JR05	0.5002	8.81	30 hours (mammalian reticulocytes, in vitro).<br><br>>20 hours (yeast, in vivo).<br><br>>10 hours (Escherichia coli, in vivo).	40.85	78.31	-0.352
	Envelope glycoprotein E	Q9J3M8	0.5340	5.26	1 hours (mammalian reticulocytes, in vitro).<br><br>30 min	36.93	73.73	-0.404

					(yeast, in vivo).<br><br>>10 hours (Escherichia coli, in vivo).			
	Envelope glycoprotein I	Q77NN4	0.4192	8.20	30 hours (mammalian reticulocytes, in vitro).<br><br>>20 hours (yeast, in vivo).<br><br>>10 hours (Escherichia coli, in vivo).	37.26	100.45	-0.022
	Envelope glycoprotein H	Q775J3	0.5123	6.16	30 hours (mammalian reticulocytes, in vitro).<br><br>>20 hours (yeast, in vivo).<br><br>>10 hours (Escherichia coli, in vivo).	33.70	96.37	0.056
	Envelope glycoprotein K	Q4JQX0	0.5731	9.01	30 hours (mammalian reticulocytes, in vitro).<br><br>>20 hours (yeast, in vivo).	40.35	106.29	0.459

					>10 hours (Escherichia coli, in vivo).			
	Envelope glycoprotein M	Q77NP2	0.4934	9.20	30 hours (mammalian reticulocytes, in vitro).<br><br>>20 hours (yeast, in vivo).<br><br>>10 hours (Escherichia coli, in vivo).	36.38	104.44	0.277

Supplementary Table S2: List of the predicted best CTL epitopes and their topology, antigenicity, allergenicity, toxicity, conservancy, and toxicity. AN; antigenicity, TT; transmembrane topology, AG; allergenicity, CN; conservancy.

**Protein name**	**Epitope**	**Start**	**End**	**AN (0.40<)**	**TT (Outside)**	**AG (Non-Allergen)**	**CN (Conserved)**	**Toxicity (Non-Toxic)**
Envelope glycoprotein B	LKDREFMPL	30	38	2.2556	Outside	Non-Allergen	Conserved	Non-Toxic
	RSAHLGDGD	33	41	1.9867	Outside	Non-Allergen	Conserved	Non-Toxic
	TYVDLNLTL	21	29	1.9634	Outside	Non-Allergen	Conserved	Non-Toxic
	VEFAMLQFT	32	40	1.8045	Outside	Non-Allergen	Conserved	Non-Toxic
	ELRDTGLLD	45	53	1.6518	Outside	Non-Allergen	Conserved	Non-Toxic
Envelope glycoprotein E	GDLNPKPQG	40	48	2.5460	Outside	Non-Allergen	Conserved	Non-Toxic
	KGDLNPKPQ	39	47	2.5286	Outside	Non-Allergen	Conserved	Non-Toxic
	DLNPKPQGQ	41	49	2.4604	Outside	Non-Allergen	Conserved	Non-Toxic
	CLGISHMEP	22	30	2.2925	Outside	Non-Allergen	Conserved	Non-Toxic
	EITPVNPGT	44	52	2.0485	Outside	Non-Allergen	Conserved	Non-Toxic
Envelope glycoprotein I	LDLRAGKSL	38	46	1.7218	Outside	Non-Allergen	Conserved	Non-Toxic
	RLCDLPATP	18	26	1.3925	Outside	Non-Allergen	Conserved	Non-Toxic
	NHVYPTDMS	11	19	1.3304	Outside	Non-Allergen	Conserved	Non-Toxic
	IKGQLVFIG	51	59	1.2003	Outside	Non-Allergen	Conserved	Non-Toxic
	KGDHVSLQV	24	32	1.0296	Outside	Non-Allergen	Conserved	Non-Toxic
Envelope glycoprotein H	VGFGIIGWM	817	825	1.9862	Outside	Non-Allergen	Conserved	Non-Toxic
	EVNIGVGFM	251	259	1.9628	Outside	Non-Allergen	Conserved	Non-Toxic
	VEVNIGVGF	250	258	1.9459	Outside	Non-Allergen	Conserved	Non-Toxic
	IKSLHWGND	63	71	1.9380	Outside	Non-Allergen	Conserved	Non-Toxic
	LIKSLHWGN	62	70	1.8897	Outside	Non-Allergen	Conserved	Non-Toxic
Envelope glycoprotein K	ALGIKTEHF	3	11	2.0611	Outside	Non-Allergen	Conserved	Non-Toxic
	GLAIKCFYI	301	309	1.6992	Outside	Non-Allergen	Conserved	Non-Toxic

	VQLKMIFFA	119	127	1.5470	Outside	Non-Allergen	Conserved	Non-Toxic
	CVQLKMIFF	118	126	1.5360	Outside	Non-Allergen	Conserved	Non-Toxic
	ISFLYMHKG	203	211	1.4966	Outside	Non-Allergen	Conserved	Non-Toxic
Envelope glycoprotein M	GPRSEKVSP	7	15	1.5474	Outside	Non-Allergen	Conserved	Non-Toxic
	AICILFVSL	244	252	1.3833	Outside	Non-Allergen	Conserved	Non-Toxic
	LLLAYKQIG	172	180	1.3278	Outside	Non-Allergen	Conserved	Non-Toxic
	WSSVSVAMS	267	275	1.2974	Outside	Non-Allergen	Conserved	Non-Toxic
	GPRSEKVSP	7	15	1.5474	Outside	Non-Allergen	Conserved	Non-Toxic

Supplementary Table S3: List of the predicted best HTL epitopes and their topology, antigenicity, allergenicity, toxicity, conservancy, and toxicity. AN; antigenicity, TT; transmembrane topology, AG; allergenicity, CN; conservancy.

Protein name	Epitope	Start	End	AN (0.40<)	TT (Outside)	AG (Non-Allergen)	CN (Conserved)	Toxicity (Non-toxic)	IFN-gamma inducing ability (Inducer)	IL-4 inducing ability (Inducer)	IL-10 inducing ability (Inducer)
Envelope glycoprotein B	DLNLTLLKDREFMPL	24	38	1.5958	Outside	Non-Allergen	Conserved	Non-toxic	Non-Inducer	Non-Inducer	Inducer
	ALLTSRLTGLALRNR	3	17	1.5466	Outside	Non-Allergen	Conserved	Non-toxic	Inducer	Non-Inducer	Inducer
	TYVDLNLTLLKDREF	21	35	1.4537	Outside	Non-Allergen	Conserved	Non-toxic	Non-Inducer	Non-Inducer	Inducer
	VDLNLTLLKDREFMP	23	37	1.4530	Outside	Non-Allergen	Conserved	Non-toxic	Non-Inducer	Inducer	Inducer
	LNLTLLKDREFMPLQ	25	39	1.4402	Outside	Non-Allergen	Conserved	Non-toxic	Non-Inducer	Non-Inducer	Inducer
Envelope glycoprotein E	YCLGISHMEPSFGLI	21	35	1.7994	Outside	Non-Allergen	Conserved	Non-toxic	Non-Inducer	Inducer	Inducer
	TAYCLGISHMEPSFG	19	33	1.7242	Outside	Non-Allergen	Conserved	Non-toxic	Non-Inducer	Inducer	Inducer
	AYCLGISHMEPSFGL	20	34	1.7198	Outside	Non-Allergen	Conserved	Non-toxic	Non-Inducer	Inducer	Inducer

	CLGISHMEPSFGLIL	22	36	1.7169	Outside	Non-Allergen	Conserved	Non-toxic	Inducer	Non-Inducer	Inducer
	LGISHMEPSFGLILH	23	37	1.4009	Outside	Non-Allergen	Conserved	Non-toxic	Inducer	Non-Inducer	Inducer
Envelope glycoprotein I	MLDLRAGKSLEDNPW	37	51	1.2576	Outside	Non-Allergen	Conserved	Non-toxic	Inducer	Inducer	Inducer
	LDLRAGKSLEDNPWL	38	52	1.2562	Outside	Non-Allergen	Conserved	Non-toxic	Inducer	Inducer	Inducer
	HMLDLRAGKSLEDNP	36	50	1.1534	Outside	Non-Allergen	Conserved	Non-toxic	Non-Inducer	Inducer	Inducer
	LLYADTVAFCFRSVQ	14	28	1.0667	Outside	Non-Allergen	Conserved	Non-toxic	Inducer	Inducer	Non-Inducer
	LYADTVAFCFRSVQV	15	29	1.0620	Outside	Non-Allergen	Conserved	Non-toxic	Non-Inducer	Inducer	Non-Inducer
Envelope glycoprotein H	LSLLFGQVKPANVDY	377	391	1.5179	Outside	Non-Allergen	Conserved	Non-toxic	Non-Inducer	Non-Inducer	Inducer
	LSPYHFKAEHRAPFP	41	55	1.4841	Outside	Non-Allergen	Conserved	Non-toxic	Inducer	Inducer	Inducer
	EHRAPFPAGRFGFLS	49	63	1.4521	Outside	Non-Allergen	Conserved	Non-toxic	Inducer	Inducer	Inducer
	KAEHRAPFPAGRFGF	47	61	1.4098	Outside	Non-Allergen	Conserved	Non-toxic	Non-Inducer	Inducer	Inducer

	SLDYRYHLSMAHTEA	273	287	1.3952	Outside	Non-Allergen	Conserved	Non-toxic	Non-Inducer	Inducer	Inducer
Envelope glycoprotein K	ALGIKTEHFIIMCLL	3	17	1.4365	Outside	Non-Allergen	Conserved	Non-toxic	Non-Inducer	Inducer	Non-Inducer
	QALGIKTEHFIIMCL	2	16	1.3810	Outside	Non-Allergen	Conserved	Non-toxic	Non-Inducer	Inducer	Non-Inducer
	GLAIKCFYIVIFAIA	1	15	1.1836	Outside	Non-Allergen	Conserved	Non-toxic	Non-Inducer	Inducer	ind
	LAIKCFYIVIFAIAV	2	16	1.1676	Outside	Non-Allergen	Conserved	Non-toxic	Non-Inducer	Inducer	Non-Inducer
	HISSGCIVLLTLGVA	43	57	1.1557	Outside	Non-Allergen	Conserved	Non-toxic	Non-Inducer	Non-Inducer	Non-Inducer
Envelope glycoprotein M	SPRLIPTTRGTLACL	332	346	1.4725	Outside	Non-Allergen	Conserved	Non-toxic	Non-Inducer	Non-Inducer	Inducer
	VVLLLAYKQIGVAAT	170	184	1.2621	Outside	Non-Allergen	Conserved	Non-toxic	Inducer	Non-Inducer	Inducer
	LIPTTRGTLACLAVF	335	349	1.2383	Outside	Non-Allergen	Conserved	Non-toxic	Non-Inducer	Non-Inducer	Inducer
	YTGIPCFYAAVVDYE	63	77	1.1469	Outside	Non-Allergen	Conserved	Non-toxic	Non-Inducer	Inducer	Non-Inducer

	AVVLLL AYKQIG VAA	169	183	1.1442	Out side	Non-Aller gen	Cons erve d	Non-toxic	Non-Induc er	Non-Induc er	Induc er

Supplementary Table S4: List of the predicted LBL epitopes and their topology, antigenicity, allergenicity, toxicity, conservancy, and toxicity. AN; antigenicity, TT; transmembrane topology, AG; allergenicity.

**Epitope**	**Start**	**End**	**AN (0.40<)**	**TT** (Outside)	**AG** (Non-Allergen)	**Toxicity (**Non-toxic)
ERGFPPTAGQPPATTKPKEITPVNPGTSP	506	534	1.0469	Outside	Non-Allergen	Non-toxic
RDELRDTGLLDYSE	703	716	1.0403	Outside	Non-Allergen	Non-toxic
LFDELELDPPEIEPG	270	284	0.9954	Outside	Non-Allergen	Non-toxic
LLSPYHFKAEHRAPFPAGRFGFLSHPVTPDVSFF	100	133	0.9314	Outside	Non-Allergen	Non-toxic
FGQVKPANVDYFISYDE	441	457	0.8483	Outside	Non-Allergen	Non-toxic
SETNEFNLNQIHLS	402	415	0.7950	Outside	Non-Allergen	Non-toxic
AFYPTGFDEELIKSLHWGND	52	71	0.6839	Outside	Non-Allergen	Non-toxic
FRSSCELISPTSY	147	159	0.5960	Outside	Non-Allergen	Non-toxic
ATIREESPPHSVVN	337	350	0.5801	Outside	Non-Allergen	Non-toxic
VTAVVSVSPSSFYESLQVEPTQSEDITRSAHLGDGDEIREAIHKSQDAET	66	115	0.5506	Outside	Non-Allergen	Non-toxic
HMLDLRAGKSLEDNPWLHEDVVTTETKSVVKEGIENHVYPTDMSTLPEKSLNDPP	216	270	0.5453	Outside	Non-Allergen	Non-toxic
GSHHNIHGVIYTSPSLQNGYSTRALFQQARLCDLPATPKGSGTSL	169	213	0.5116	Outside	Non-Allergen	Non-toxic
PLTTKGLKQLPEGMDPFAEKPNATDTPIEEIGDSQNTEPSVNSGFDPDKFRE	823	874	0.4332	Outside	Non-Allergen	Non-toxic

Supplementary Table S5: Epitopes used in vaccine construction

CTL Epitopes	HTL Epitopes	LBL Epitopes
LKDREFMPL	DLNLTLLKDREFMPL	ERGFPPTAGQPPATTKPKEITPVNPGTSP
GDLNPKPQG	YCLGISHMEPSFGLI	LFDELELDPPEIEPG
LDLRAGKSL	MLDLRAGKSLEDNPW	FGQVKPANVDYFISYDE
VEVNIGVGF	LSLLFGQVKPANVDY	SETNEFNLNQIHLS
GLAIKCFYI	ALGIKTEHFIIMCLL	FRSSCELISPTSY
GPRSEKVSP	VVLLLAYKQIGVAAT	ATIREESPPHSVVN
TYVDLNLTL	ALLTSRLTGLALRNR	
CLGISHMEP	AYCLGISHMEPSFGL	
RLCDLPATP	LLYADTVAFCFRSVQ	
IKSLHWGND	LSPYHFKAEHRAPFP	
ALGIKTEHF	GLAIKCFYIVIFAIA	
AICILFVSL	LIPTTRGTLACLAVF	

Supplementary Table S6: Tools and servers used in epitope prediction with their specific application, input and output.

Tool name	Application	Input	Output
VaxiJen v2.0	Determine the antigenicity	Amino acid sequence (Epitope/Protein/Vaccine)	Antigenic score
TMHMM 2.0	Determine the transmembrane topology	Amino acid sequence (Epitope/Protein/Vaccine)	Location of segments (inside/outside)
AllergenFP	Determine the Allergenecity	Amino acid sequence (Epitope/Protein/Vaccine)	Allergen/Non-allergen
AllerTOP	Determine the Allergenecity	Amino acid sequence (Epitope/Protein/Vaccine)	Allergen/Non-allergen
AlgPred2.0	Determine the Allergenecity	Amino acid sequence (Epitope/Protein/Vaccine)	Allergen/Non-allergen
ToxinPred	Determine the toxicity	Amino acid sequence (Epitope/Protein/Vaccine)	Toxic/Nontoxic
IFNepitope	Determine the capacity of to induce IFN-γ	Amino acid sequence (Epitope)	Inducer/Non-inducer
IL4pred	Determine the capacity of to induce IL-4	Amino acid sequence (Epitope)	Inducer/Non-inducer
IL10pred	Determine the capacity of to induce IL-10	Amino acid sequence (Epitope)	Inducer/Non-inducer
IEDB epitope conservancy tool	Determine the conservancy among targeted strain	Amino acid sequence (Epitope)	Conservancy (%)

Supplementary Table S7: Antigenicity, Allergenecity, solubility, and biophysical properties of Vaccine construct 1 (V1) and Vaccine construct 2 (V2).

Features	V1	V2
**Antigenicity (0.40<)**	0.9833 (Antigenic)	0.8561 (Antigenic)
**Allergenecity**	Non-Allergen	Non-Allergen
**Solubility (0.45<)**	0.507 (Soluble)	0.484 (Soluble)
**Number of amino acids**	322	302
**Theoretical Isoelectric point (pI) (4<)**	9.18	9.47
**Formula**	$C_{1557}H_{2456}N_{410}O_{432}S_{14}$	$C_{1460}H_{2293}N_{399}O_{395}S_{16}$
**Total number of atoms**	4869	4563
**(Asp + Glu)**	29	16
**(Arg + Lys)**	41	35
**Half-life**	30 hours (mammalian reticulocytes, in vitro).<br>>20 hours (yeast, in vivo).<br>>10 hours (Escherichia coli, in vivo).	30 hours (mammalian reticulocytes, in vitro).<br>>20 hours (yeast, in vivo).<br>>10 hours (Escherichia coli, in vivo).
**Aliphatic index (60<)**	81.89	85.13
**Instability index (40>)**	31.42	36.12

**GRAVY**	-0.169	0.060

Supplementary Table S8: Secondary structure analysis of Vaccine Construct 1 (V2) and Vaccine Construct 2 (V2) predicted by SOPMA server

Parameters	V1	V2
Alpha Helix	18.63%	29.47%
Extended Strand	23.29%	23.18%
Beta Turn	8.07%	3.31%
Random Coil	50.00%	44.04%

Supplementary Table S9: Docking result between best CTL epitopes with HLA-A*02:01 and best HTL epitopes with HLA-DRB1*15

HLA Allele	Epitope	Docking score
HLA-A*02:01 (PDB ID: 4U6X)	GDLNPKPQG	-130.74
	GLAIKCFYI	-184.74
	GPRSEKVSP	-154.80
	LDLRAGKSL	-147.20
	LKDREFMPL	-156.80
	VEVNIGVGF	-147.56
HLA-DRB1*15 (PDB ID: 5V4M)	ALGIKTEHFIIMCLL	-208.12
	DLNLTLLKDREFMPL	-190.38
	LSLLFGQVKPANVDY	-198.15
	MLDLRAGKSLEDNPW	-217.37
	VVLLLAYKQIGVAAT	-215.30
	YCLGISHMEPSFGLI	-220.98

Supplementary Figures

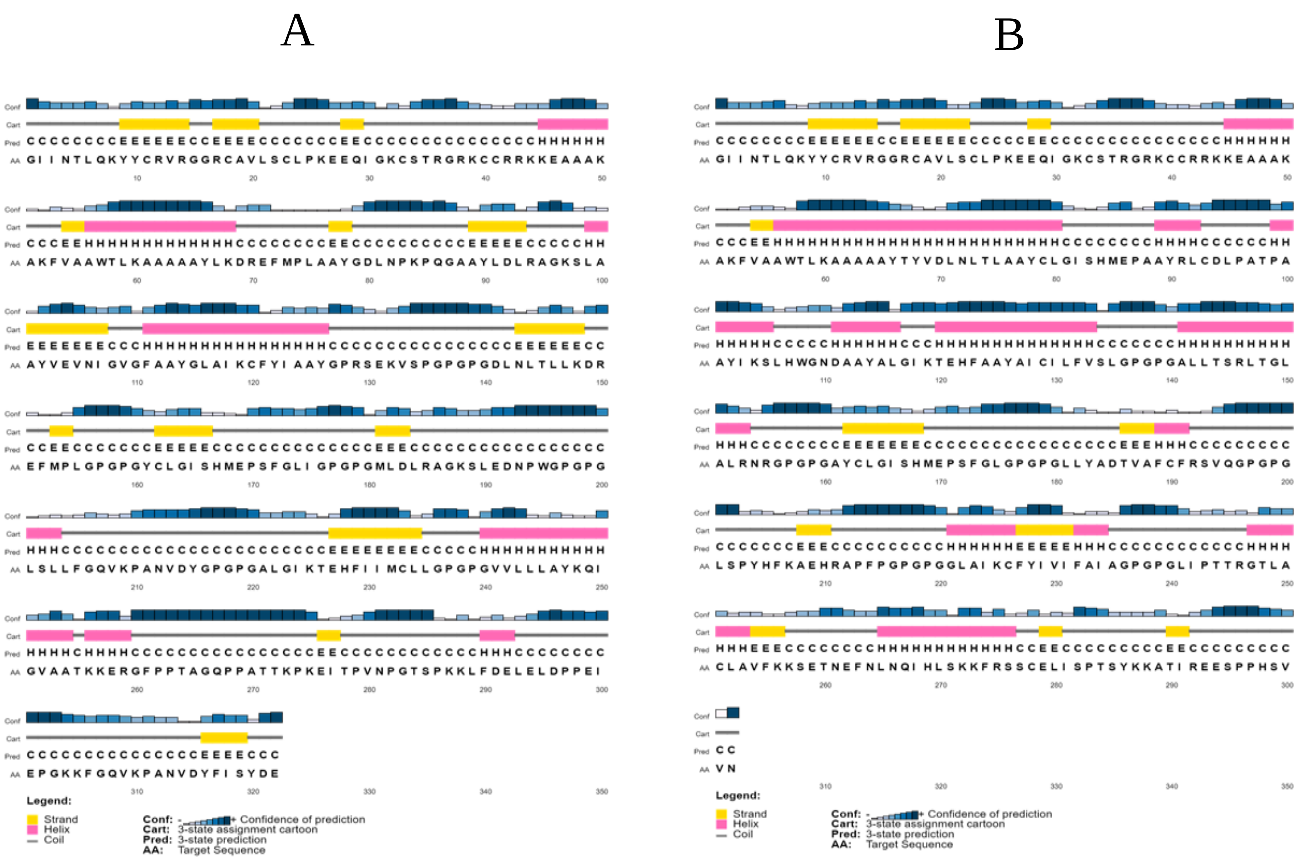

Supplementary Figure S1: Secondary Structure of A) Vaccine construct 1, B) Vaccine construct 2

A B

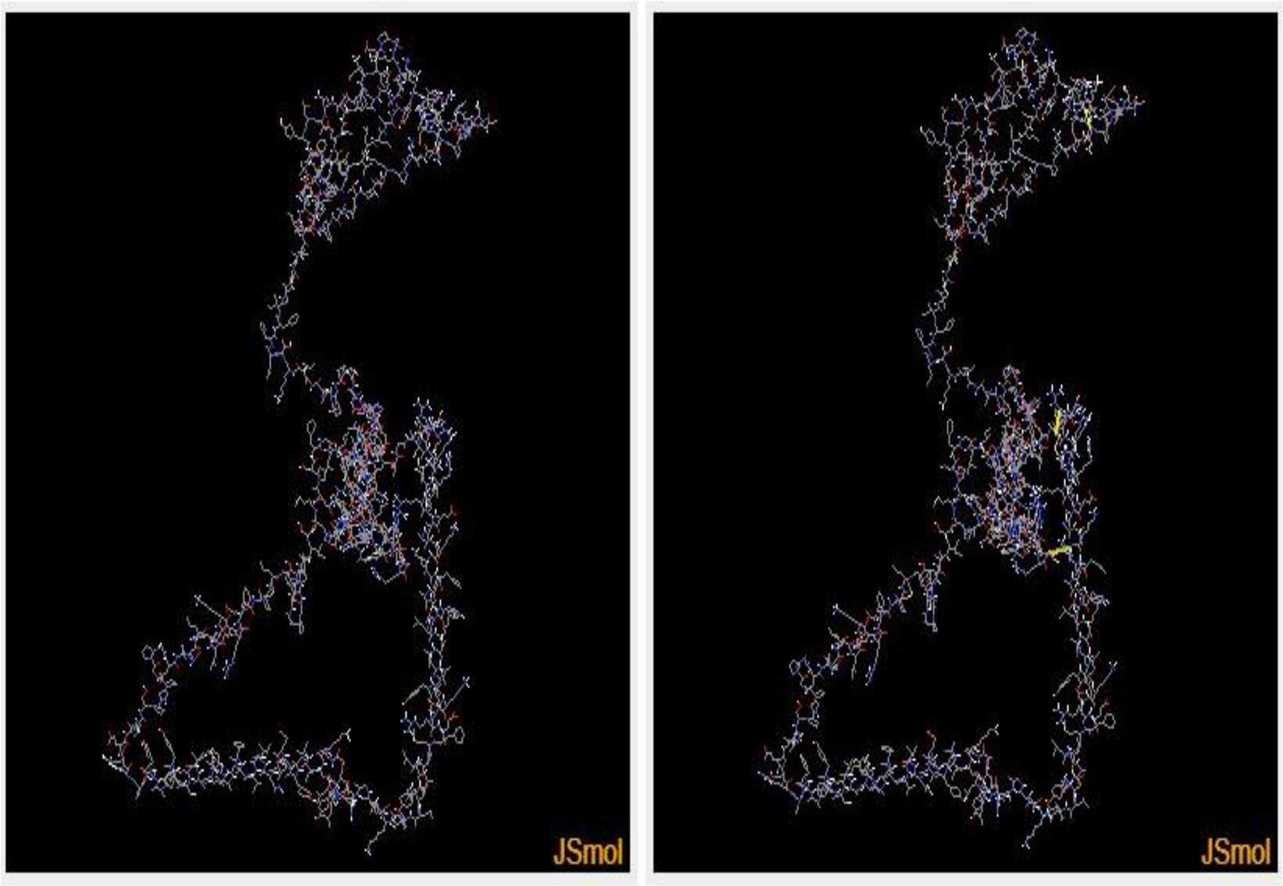

Supplementary Figure S2: Disulfide Engineering of vaccine construct 1 A) Original Model, B) Mutant Model

A B

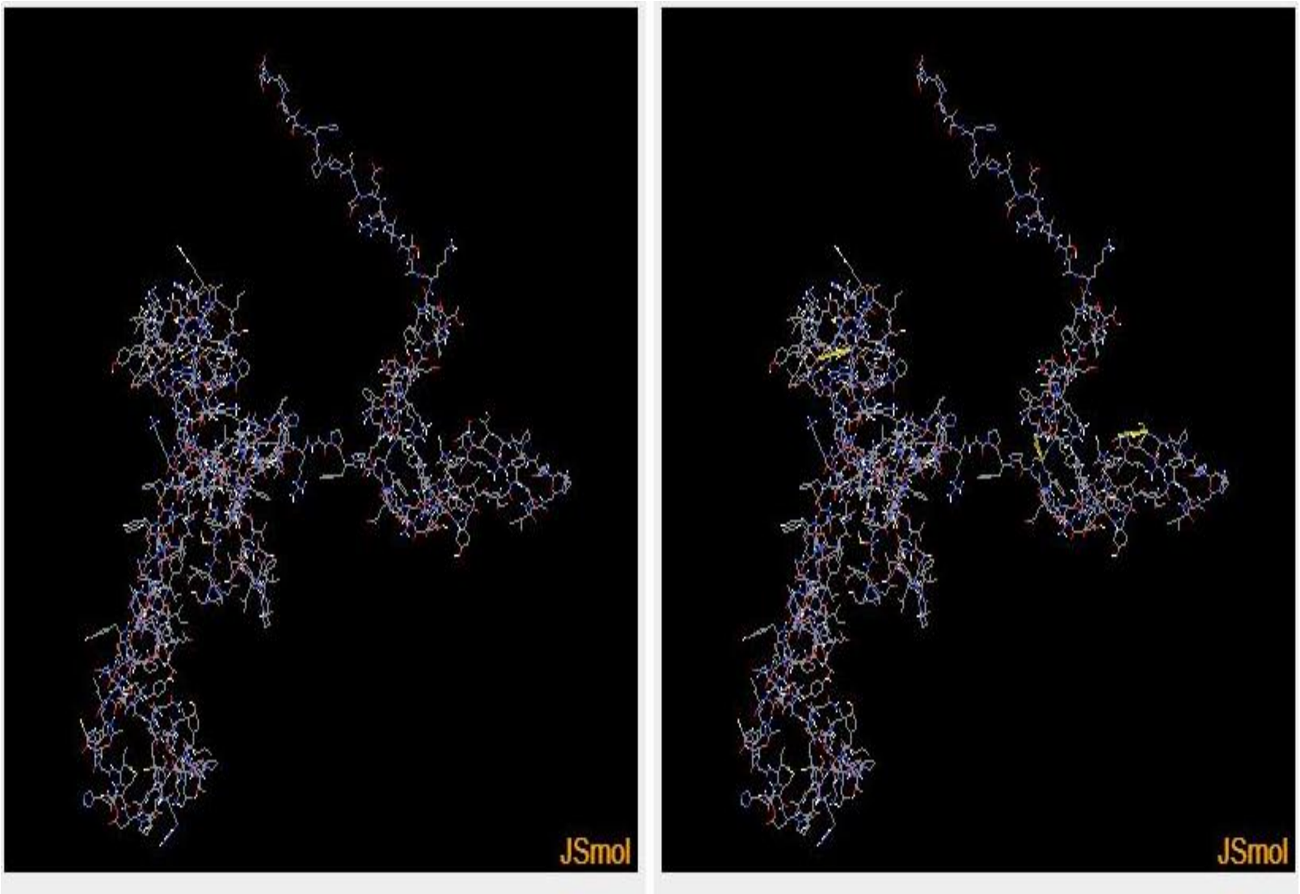

Supplementary Figure S3: Disulfide Engineering of vaccine construct 2 A) Original Model, B) Mutant Model

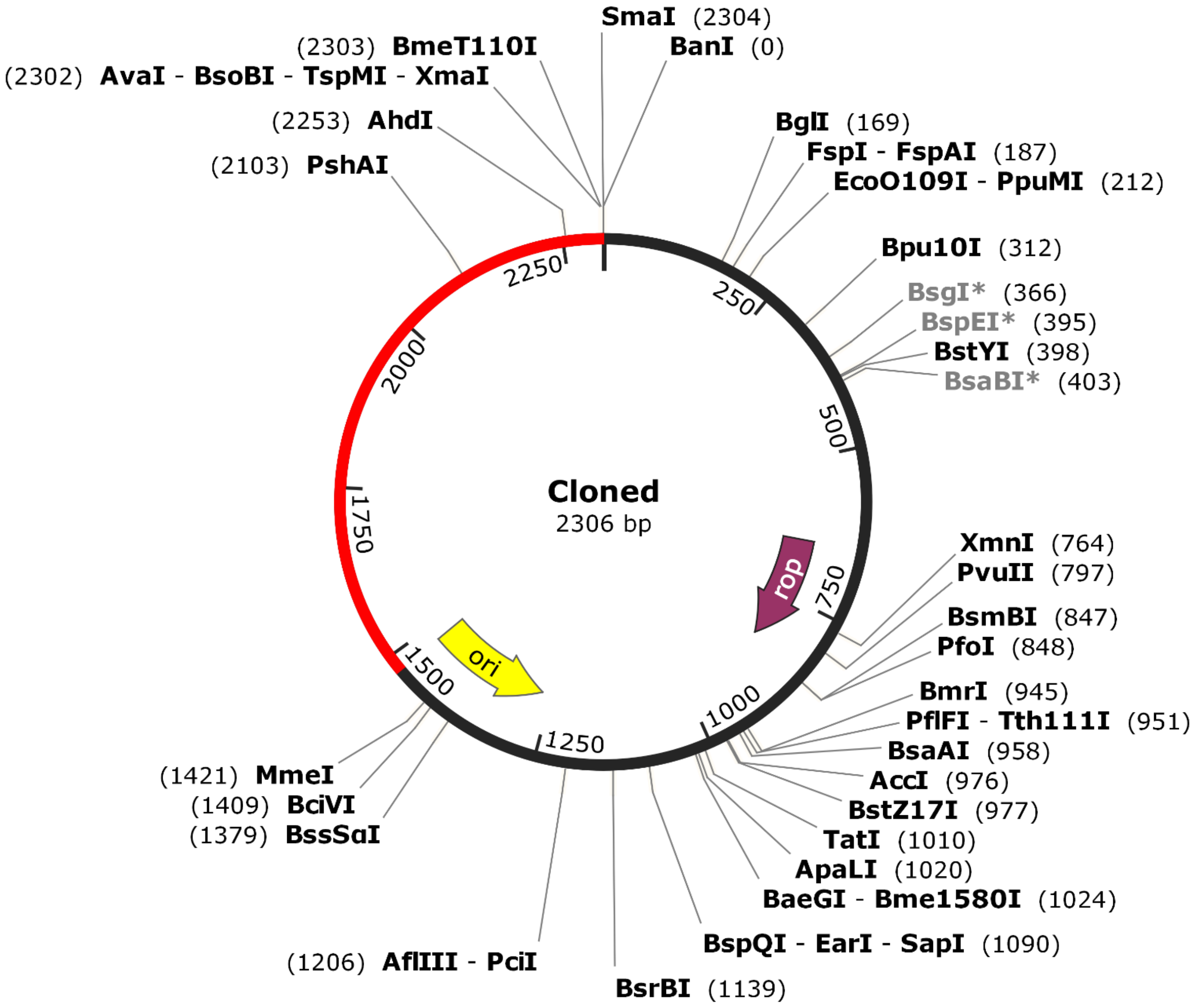

Supplementary Figure S4: The recombinant plasmid designed for mass production of the VZV vaccine.
